# Re-annotation of the CAZy genes of *Trichoderma reesei* and transcription in the presence of lignocellulosic substrates

**DOI:** 10.1186/1475-2859-11-134

**Published:** 2012-10-04

**Authors:** Mari Häkkinen, Mikko Arvas, Merja Oja, Nina Aro, Merja Penttilä, Markku Saloheimo, Tiina M Pakula

**Affiliations:** 1 VTT Technical Research Centre of Finland, P.O.Box 1000, Tietotie 2, Espoo, FI-02044, VTT, Finland

**Keywords:** Carbohydrate active enzymes, Cellulase, Hemicellulase, Lignocellulose, Transcriptome, Transcriptional profiling, Gene regulation, Wheat, Spruce, Bagasse, Biorefinery

## Abstract

**Background:**

*Trichoderma reesei* is a soft rot Ascomycota fungus utilised for industrial production of secreted enzymes, especially lignocellulose degrading enzymes. About 30 carbohydrate active enzymes (CAZymes) of *T. reesei* have been biochemically characterised. Genome sequencing has revealed a large number of novel candidates for CAZymes, thus increasing the potential for identification of enzymes with novel activities and properties. Plenty of data exists on the carbon source dependent regulation of the characterised hydrolytic genes. However, information on the expression of the novel CAZyme genes, especially on complex biomass material, is very limited.

**Results:**

In this study, the CAZyme gene content of the *T. reesei* genome was updated and the annotations of the genes refined using both computational and manual approaches. Phylogenetic analysis was done to assist the annotation and to identify functionally diversified CAZymes. The analyses identified 201 glycoside hydrolase genes, 22 carbohydrate esterase genes and five polysaccharide lyase genes. Updated or novel functional predictions were assigned to 44 genes, and the phylogenetic analysis indicated further functional diversification within enzyme families or groups of enzymes. GH3 β-glucosidases, GH27 α-galactosidases and GH18 chitinases were especially functionally diverse. The expression of the lignocellulose degrading enzyme system of *T. reesei* was studied by cultivating the fungus in the presence of different inducing substrates and by subjecting the cultures to transcriptional profiling. The substrates included both defined and complex lignocellulose related materials, such as pretreated bagasse, wheat straw, spruce, xylan, Avicel cellulose and sophorose. The analysis revealed co-regulated groups of CAZyme genes, such as genes induced in all the conditions studied and also genes induced preferentially by a certain set of substrates.

**Conclusions:**

In this study, the CAZyme content of the *T. reesei* genome was updated, the discrepancies between the different genome versions and published literature were removed and the annotation of many of the genes was refined. Expression analysis of the genes gave information on the enzyme activities potentially induced by the presence of the different substrates. Comparison of the expression profiles of the CAZyme genes under the different conditions identified co-regulated groups of genes, suggesting common regulatory mechanisms for the gene groups.

## Background

Natural resources are diminishing while the demand for commodities, energy and food increases. This sets a requirement to find solutions for efficient utilisation of renewable biological material in production of energy and chemicals. The most abundant terrestrial renewable organic resource is lignocellulose that can be derived from industrial side-streams, municipal waste, or by-products of agriculture and forestry, and can be used as a raw material in biorefinery applications (for reviews, see [1] and [2]). It consists of cellulose, hemicellulose and lignin. Cellulose is a polymer of β-1,4-linked D-glucose units. Hemicelluloses are more heterogeneous materials that can be classified as xylans, mannans, xyloglucans and mixed-linkage glucans according to the main sugar units forming the backbone. In addition, hemicelluloses are often branched and contain side chains such as galactose, arabinan, glucuronic acid and acetyl groups. Lignin is a very resistant material that lacks a precise structure and consists of aromatic building-blocks [3]. Typically, conversion of biomass raw material includes physical and chemical pre-treatment followed by enzymatic hydrolysis of the polymers into monosaccharides and fermentation of the sugars by micro-organisms to produce higher value products, such as transport fuels and chemical feedstocks [1]. Due to the complex and heterogeneous nature of the material, both the pre-treatment method and the enzyme composition need to be adjusted according to the type of the raw material. Since the cost of enzymes is still a major limitation in the utilisation of biomass, improvement of the enzyme production systems and use of optimal mixtures of synergistic enzymes, as well as the choice of raw material and pretreatment method, are of importance in setting up a cost effective biorefinery process [4].

The CAZy database [5,6] contains information on carbohydrate active enzymes involved in breakdown, modification and synthesis of glycosidic bonds. Enzyme classes covered by CAZy classification include glycoside hydrolases (GH), carbohydrate esterases (CE), polysaccharide lyases (PL) and glycosyltransferases (GT). In addition, enzymes containing a carbohydrate binding module (CBM) are also covered. Enzymes are further classified into different families, originally based on hydrophobic cluster analysis and later on utilising sequence similarity analyses supplemented by structural information together with experimental evidence available in scientific literature. Enzymes degrading lignocellulosic plant cell wall material are known to be especially abundant in glycoside hydrolase and carbohydrate esterase families.

*Trichoderma reesei* (anamorph of *Hypocrea jecorina*), a mesophilic soft-rot of the phylum Ascomycota, is known for its ability to produce high amounts of cellulases and hemicellulases. It is widely employed to produce enzymes for applications in pulp and paper, food, feed and textile industries and, currently, with increasing importance in biorefining [7]. *T. reesei* produces two exo-acting cellobiohydrolases (CBHI/CEL7A and CBHII/CEL6A) [8-10]. Five endo-acting cellulases (EGI/CEL7B, EGII/CEL5A, EGIII/CEL12A, EGIV/CEL61A, EGV/CEL45A) [11-15] have been characterized, and three putative endoglucanases (CEL74A, CEL61B and CEL5B) have been found using cDNA sequencing [16]. From these enzymes CEL74A has been later characterized as a putative xyloglucanase [17], and enzymes of the glycoside hydrolase family GH61 have been shown to enhance lignocellulose degradation by an oxidative mechanism [18]. The *T. reesei* genome also encodes two characterized β-glucosidases (BGLI/CEL3A and BGLII/CEL1A) [19-22] and five predicted β-glucosidases (CEL3B, CEL3D, CEL1B, CEL3C, CEL3E) [16]. β-glucosidases hydrolyse non-reducing β-D-glucosyl residues in oligomeric cellulose degradation products and carry out transglycosylation reactions of them. Swollenin (SWOI) participates in the degradation of biomass by disrupting crystalline cellulose structure without apparent release of sugars [23]. Hemicellulases produced by *T. reesei* include four xylanases (XYNI, XYNII, XYNIII and XYNIV) [24-26], a mannanase (MANI) [27], one characterized (AXEI) [28] and one predicted (AXEII) acetyl xylan esterase [16], α-glucuronidase (GLRI) [29], one characterized (ABFI) [30] and two predicted (ABFII, ABFIII) arabinofuranosidases [16,31], three α-galactosidases (AGLI, AGLII and AGLIII) [32,33] as well as a β-xylosidase (BXLI) [30] that digests oligosaccharides derived from xylan. An acetyl esterase gene (AESI) that removes acetyl groups from hemicellulose has also been identified [34]. Glucuronoyl esterase CIPII is believed to participate in the degradation of lignocellulose biomass by cleaving ester linkages between lignin and hemicellulose and so facilitating the removal of lignin [16,35,36]. In addition to the characterized genes, several novel candidate lignocellulose degrading enzymes have been identified from the genome of *T. reesei* based on conserved domains and homology to enzymes from other fungi [37,38].

The majority of the characterised cellulase and hemicellulase genes of *T. reesei* are regulated by the type of carbon source available, in order to ensure production of hydrolytic enzymes required for degradation of the substrate and, on the other hand, to avoid energy consuming enzyme production under conditions where easily metabolisable carbon source is available. In most cases, the genes encoding the hydrolytic enzymes are repressed by glucose, and induced by various compounds derived from plant cell wall material (for reviews, see [39-41]) or their metabolic derivatives. Cellulase and hemicellulase genes have been shown to be induced e.g. in the presence of cellulose, β-glucan, xylans and a variety of mono- and disaccharides, such as lactose, cellobiose, sophorose, L-sorbose, L-arabitol, xylobiose, cellobiose and galactose, different sets of genes being induced by the different compounds [16,42-46]. However, information on the co-induced gene groups on different substrates is still rather limited, especially regarding the novel candidate glycoside hydrolase genes identified from the genome sequence.

In this study, we have used computational methods and phylogenetic analysis to update the annotation and functional prediction of the CAZymes of *T. reesei.* Furthermore, we have analysed the expression of the CAZyme genes of *T. reesei* in the presence of different lignocellulose materials as inducing substrates in order to identify co-regulated gene groups, and to get information on the enzyme activities induced by the presence of the substrates. The selected substrates included both purified compounds and polymeric carbohydrates as well as complex lignocellulosic raw materials. The information was used to identify co-regulated gene groups and sets of genes induced by the substrates.

## Results

### Identification of *T. reesei* CAZyme genes

We have updated the existing annotations of CAZyme genes of *T. reesei* using computational methods and manual proofreading in order to remove discrepancies between annotations in the different genome versions [38,47] and published literature. We have also used comparative genomics and phylogenetic information to update and refine functional prediction of the encoded enzymes. The initial set of candidates for *T. reesei* CAZymes was identified by mapping the *T. reesei* proteome to CAZy database [5,6] using blastp [48], and a preliminary function prediction was assigned for the candidates based on the homologues (Additional file 1). After removal of candidates with blast hits that had apparently incorrect annotation and genes with other function predictions not related to CAZy, a total of 387 candidate genes were retrieved. Glycosyltransferase genes (99 GT genes) and the carbohydrate esterase family 10 genes (31 CE genes) were excluded from the further study since these genes mostly encode activities not involved in degradation of plant cell wall material. Furthermore, the CE10 family is no longer updated in the CAZy database. In order to verify whether the selection of candidate genes for CAZymes was supported by protein sequences from other fungi, the protein homology clusters described in [49] and updated to include 49 fungal species [50], were mapped to the CAZy database. The homology clusters were then filtered based on the average sequence identity percentage and length of the blast alignment with CAZymes (Additional file 2B and C). The clusters containing CAZymes were manually reviewed for consistency of protein domain content and quality of gene models to get reliable candidates. In addition, a few genes not fulfilling all the computational criteria were included in the further study. The proteins encoded by *T. reesei* genes 59791 and 73101 were found in protein homology clusters with no other members. The phylogeny of these two genes is discussed later. The gene 22129 was included in the study due to its previous annotation as distantly related to GH61 family [38]. In total 228 CAZyme genes remained after the computational and manual filtering.

### Functional diversification of *T. reesei* CAZyme genes

CAZy family membership was assigned to the *T. reesei* gene products based on the CAZy family members of other species in the same protein homology cluster (clustering of the 49 fungal species) by majority vote. The homology clusters typically corresponded to groups of orthologous gene products supplemented with paralogues derived from gene duplications that have occurred in some sub lineage of the 49 species. *T. reesei* CAZymes were predicted to belong to 61 CAZy families (excluding CE10). The members of 27 CAZy families were divided into more than one protein homology clusters (Table 1). The fact that a family was divided into several clusters was interpreted as a sign of functional diversification within the family. In *Saccharomyces cerevisiae* most duplicated genes are derived from the genome duplication event that took place approximately 100 million years ago [51,52]. Recently, it has been experimentally shown that these duplicates have diverged in cellular, if not molecular, functions [53]. Sordariomycetes diverged from other fungi roughly 400 million years ago hence it is safe to assume that duplicate genes that were likely to exist already in the common ancestor of Sordariomycetes have had ample time to diverge functionally [54]. Thus, each protein homology cluster with multiple *T. reesei* CAZymes was searched for signs of further functional diversification within the cluster. If the phylogenetic analysis of a homology cluster suggested that the gene duplication predated the common ancestor of Sordariomycetes, it was interpreted as a sign of functional diversification between the *T. reesei* gene duplicates (Table 1).

**Table 1:** **CAZyme genes of *T. reesei***

**Gene ID**^**a**^	**Name**^**b**^	**CAZy**^**c**^	**Annotation**	**CBM module**^**d**^	**Ref**^**e**^	**Prot. cluster**^**f**^	**Functional subgroup**^**g**^
**A.**							
107268		CE1	Cand. S-formylglutathione hydrolase		This study	2607	2607a
72072		CE1	Cand. esterase		[38]	6197	6197a
107850		CE1	Cand. esterase		[47]	6197	6197a
44366		CE3	Cand. esterase		This study	2806	2806a
70021		CE3	Cand. acetyl xylan esterase		[38]	2806	2806a
41248		CE3	Cand. acetyl xylan esterase		[47]	6113	6113a
107488		CE3	Cand. esterase		This study	6113	6113a
31227		CE3	Cand. esterase/suberinase		This study	13836	13836a
67678		CE4	Cand. chitin deacetylase	CBM18	[37]	302	302a
69490		CE4	Cand. chitin deacetylase	CBM18	[37]	302	302b
65215		CE4	Cand. imidase		[38]	2445	2445a
105072		CE4	Cand. polysaccharide deacetylase		[38], A	4972	4972a
60489		CE5	Cand. cutinase		[38]	540	540a
44214	axe2	CE5	Cand. acetyl xylan esterase		[16]	865	865a
54219		CE5	Cand. acetyl xylan esterase		[31]	865	865b
73632	axe1	CE5	Acetyl xylan esterase	CBM1	[28]	865	865b
79671		CE9	Cand. N-acetyl-glucosamine-6-phosphate deacetylase		[38]	2516	2516a
3101		CE14	Cand. N-acetylglucosaminylphosphatidylinositol de-N-acetylase		This study	2076	2076a
58550		CE14	Cand. N-acetylglucosaminylphosphatidylinositol de-N-acetylase		This study	7267	7267a
123940	cip2	CE15	Glucuronoyl esterase		[16]	4243	4243a
103825		CE16	Cand. acetyl esterase		This study	3851	3851a
121418	aes1	CE16	Acetyl esterase		[34]	3851	3851a
22197	cel1b	GH1	Cand. β-glucosidase		[16]	662	662a
120749	bgl2/cel1a	GH1	β-glucosidase		[21]	662	662b
5836		GH2	Cand. β-mannosidase		[38]	609	609a
59689		GH2	Cand. β-mannosidase		[38]	609	609a
69245		GH2	Cand. β-mannosidase		[38]	609	609a
62166		GH2	Cand. β-mannosidase		[38]	609	609b
57857		GH2	Cand. β-mannosidase		[38], A	609	609c
77299	gls93	GH2	Exo-β-D-glucosaminidase		[83]	2917	2917a
76852		GH2	Cand. β-galactosidase/β-glucuronidase		[38], A	4433	4433a
102909		GH2	Cand. GH2 protein		This study	125855	125855a
121735	cel3b	GH3	Cand. β-glucosidase		[16]	110	110a
47268	bgl3i	GH3	Cand. β-glucosidase		[62]	110	110b
66832		GH3	Cand. β-glucosidase		[37]	110	110c
76227	cel3e	GH3	Cand. β-glucosidase		[16]	110	110d
76672	bgl1/cel3a	GH3	β-glucosidase		[22]	110	110e
104797	bgl3j	GH3	Cand. β-glucosidase		[62]	110	110f
46816	cel3d	GH3	Cand. β-glucosidase		[16]	132	132a
82227	cel3c	GH3	Cand. β-glucosidase		[16]	132	132b
108671	bgl3f	GH3	Cand. β-glucosidase/glucan 1,4-β-glucosidase		[62], A	132	132c
69557		GH3	Cand. β-N-acetylglucosaminidase		[38], A	2317	2317a
79669		GH3	Cand. β-N-acetylglucosaminidase		[38], A	2317	2317b
58450	xyl3b	GH3	Cand. β-xylosidase		[62]	3274	3274a
121127	bxl1	GH3	β-xylosidase		[30]	3274	3274b
64375		GH5	Cand. glucan β-1,3-glucosidase		[38]	173	173a
64906		GH5	Cand. endo-β-1,6-glucanase		[38]	173	173b
77506	cel5d	GH5	Cand. β-glycosidase		[62]	732	732a
82616	cel5b	GH5	Cand. membrane bound endoglucanase		[16]	778	778a
120312	egl2/cel5a	GH5	Endo-β-1,4-glucanase	CBM1	[15]	778	778a
56996	man1	GH5	β-Mannanase	CBM1	[27]	1854	1854a
53731		GH5	Cand. endo-β-1,4-glucanase		[37]	8196	8196a
71554		GH5	Cand. β-1,3-mannanase/endo-β-1,4-mannosidase		[38], A	16589	16589a
72567	cbh2/cel6a	GH6	Cellobiohydrolase	CBM1	[10]	2966	2966a
122081	egl1/cel7b	GH7	Endo-β-1,4-glucanase	CBM1	[11]	470	470a
123989	cbh1/cel7a	GH7	Cellobiohydrolase	CBM1	[8]	470	470b
120229	xyn3	GH10	Endo-β-1,4-xylanase		[26]	429	429a
74223	xyn1	GH11	Endo-β-1,4-xylanase		[24]	643	643a
112392	xyn5	GH11	Cand. endo-β-1,4-xylanase		[58]	643	643a
123818	xyn2	GH11	Endo-β-1,4-xylanase		[24]	643	643b
123232	egl3/cel12a	GH12	Endo-β-1,4-glucanase		[12]	1708	1708a
77284		GH12	Cand. endo-β-1,4-glucanase		[38], A	7857	7857a
59578		GH13	Cand. α-glucosidase		[38]	316	316a
108477		GH13	Cand. α-glucosidase/oligo α-glucosidase		[38]	316	316b
105956		GH13	Cand. α-amylase		[38]	394	394a
57128		GH13	Cand. glycogen debranching enzyme		[38]	1465	1465a
123368		GH13	Cand. 1,4-α-glucan branching enzyme		[38]	1522	1522a
**B.**							
1885	gla	GH15	Glucoamylase		[84]	608	608a
65333		GH15	Cand. α-glycosidase (Glucoamylase and related glycosyl hydrolases)		[38], A	3601	3601a
121294		GH16	Cand. glucan endo-1,3(4)-β-D-glucosidase		[38]	169	169a
38536		GH16	Cand. glucan endo-1,3(4)-β-D-glucosidase		[38]	169	169b
49274		GH16	Cand. glucan endo-1,3(4)-β-D-glucosidase		[37]	169	169c
55886		GH16	Cand. glucan endo-1,3(4)-β-D-glucosidase		[38], A	169	169c
41768		GH16	Cand. cell wall glucanosyltransferase		[37], A	248	248
58239		GH16	Cand. cell wall glucanosyltransferase		[37], A	248	248a
66843		GH16	Cand. cell wall glucanosyltransferase		[47], A	248	248b
65406		GH16	Cand. cell wall glucanosyltransferase		[37], A	248	248c
50215		GH16	Cand. endo-1,3-β-D-glucosidase/1,3-glucan binding protein		[38], A	1176	1176a
76266		GH16	Cand. cell wall glucanosyltransferase	CBM18	[38], A	2089	2089a
122511		GH16	Cand. glucan endo-1,3(4)-β-D-glucosidase		[38]	2996	2996a
123726		GH16	Cand. glucan endo-1,3(4)-β-D-glucosidase		[38]	2996	2996a
39755		GH16	Cand. glucan endo-1,3(4)-β-D-glucosidase		[38], A	3545	3545a
70542		GH16	Cand. b-glycosidase (endo-beta-1,3(4)-β-D-glucanase)		[38], A	6108	6108a
71399		GH16	Cand. endo-1,3-β-glucanase		[38], A	9096	9096a
73101		GH16	Cand. glucan endo-1,3-1,4-β-D-glucosidase		[38], A	125817	125817a
49193		GH17	Cand. glucan 1,3-β-glucosidase		[38], A	536	536a
76700		GH17	Cand. glucan 1,3 β-glucosidase/ glucan endo-1,3-β-glucosidase		[38], A	2531	2531a
39942		GH17	Cand. glucan endo-1,3-β-glucosidase		[38]	2892	2892a
66792		GH17	Cand. glucan endo-1,3-β-glucosidase		[38], A	2892	2892b
59082	chi18-2	GH18	Cand. chitinase		[59]	115	115a
62704	chi18-3	GH18	Cand. chitinase		[59]	115	115a
2735	chi18-6	GH18	Cand. chitinase		[59]	115	115b
80833	chi46	GH18	Chitinase		[85]	115	115b
81598	chi18-7	GH18	Cand. chitinase		[59]	115	115c
56894	chi18-10	GH18	Cand. chitinase	CBM18	[59]	200	200a
108346	chi18-8	GH18	Cand. chitinase	CBM18	[59]	200	200b
53949	chi18-1	GH18	Cand. chitinase	CBM18	[59]	200	200c
72339	chi18-9	GH18	Cand. chitinase	CBM18	[59]	200	200d
119859	chi18-13	GH18	Cand. chitinase		[59]	410	410
43873	chi18-12	GH18	Cand. chitinase		[59]	410	410a
110317	chi18-17	GH18	Cand. chitinase	CBM1	[59]	410	410a
68347	chi18-16	GH18	Cand. chitinase	CBM1	[59]	410	410b
124043	chi18-14	GH18	Cand. chitinase	CBM1	[59]	410	410b
66041	chi18-18	GH18	Cand. chitinase		[59]	410	410c
65162	Endo T	GH18	Endo-N-acetyl-β-D-glucosaminidase		[86]	3445	3445a
121355	chi18-rel2	GH18	Cand. Endo-N-acetyl-β-D-glucosaminidase		[38], A	3445	3445a
56448	chi18-11	GH18	Cand. chitinase		[59]	3553	3553a
62645	chi18-4	GH18	Cand. chitinase		[59]	3553	3553a
59791	chi18-15	GH18	Cand. chitinase		[59]	125792	125792a
21725		GH20	Cand. exochitinase		[38]	1025	1025a
23346	nag2	GH20	Cand. exochitinase		[38]	1025	1025a
105931		GH20	Cand. N-acetyl-β-hexosaminidase		[38]	4197	4197a
109278		GH24	Cand. lysozyme		[38]	3728	3728a
103458		GH25	Cand. N,O-diacetylmuramidase		[38]	5086	5086a
55999		GH27	Cand. α-galactosidase		[58]	617	617a
65986		GH27	Cand. α-galactosidase		[38]	617	617a
72632	agl1	GH27	α-galactosidase		[32]	617	617b
27219		GH27	Cand. α-galactosidase		[38]	12458	12458a
27259		GH27	Cand. α-galactosidase		[38]	12458	12458a
59391		GH27	Cand. α-galactosidase		[38]	12458	12458a
72704	agl3	GH27	α-galactosidase		[32]	12458	12458a
75015		GH27	Cand. α-galactosidase		[38]	12458	12458a
70186		GH28	Cand. polygalacturonase/xylogalacturonan hydrolase		[38]	295	295a
112140	pgx1	GH28	Cand. exo-polygalacturonase		[58]	295	295b
122780	rgx1	GH28	Cand. exo-rhamnogalacturonase		[58]	295	295c
103049		GH28	Cand. endo-polygalacturonase		[38]	870	870a
69276		GH30	Cand. endo-β-1,4-xylanase		[38]	6176	6176a
111849	xyn4	GH30	Endo-β-1,4-xylanase		[87]	6176	6176a
3094		GH30	Cand. glucan endo 1,6-β-glucanase		[38]	7813	7813a
69736		GH30	Cand. glucan endo 1,6-β-glucanase		[38]	7813	7813a
110894		GH30	Cand. endo-β-1,6-galactanase		[38]	16621	16621a
82235		GH31	Cand. α-glucosidase		[38]	594	594a
121351	gls2	GH31	Glucosidase II alpha subunit		[88]	1379	1379a
69944		GH31	Cand. α-xylosidase/α-glucosidase		[38]	3846	3846a
60085		GH31	Cand. α-glucosidase		[38]	5176	5176a
80240	bga1	GH35	β-galactosidase		[89]	675	675a
64827		GH36	Cand. raffinose synthase domain protein		[38], A	3772	3772a
124016	agl2	GH36	α-galactosidase		[32]	4771	4771a
120676		GH37	Cand. α,α-trehalase		[38]	769	769a
123226		GH37	Cand. α,α-trehalase		[38]	4723	4723a
3196		GH38	Cand. α-mannosidase		[38]	1337	1337a
73102		GH39	Cand. β-xylosidase		[38], A	4388	4388a
3739		GH43	Cand. β-xylosidase/α-L-arabinofuranosidase		[38]	586	586a
68064		GH43	Cand. β-xylosidase/α-L-arabinofuranosidase		[47]	7306	7306a
49976	egl5/cel45a	GH45	Endo-β-1,4-glucanase	CBM1	[14]	12601	12601a
**C.**							
45717	mds1	GH47	α-1,2-mannosidase		[90]	70	70a
2662		GH47	Cand. α-1,2-mannosidase		[38]	70	70b
111953		GH47	Cand. α-1,2-mannosidase		[38]	70	70c
79960		GH47	Cand. α-1,2-mannosidase		[47]	70	70d
65380		GH47	Cand. α-1,2-mannosidase		[38]	70	70e
79044		GH47	Cand. α-1,2-mannosidase		[38]	70	70e
22252		GH47	Cand. α-1,2-mannosidase		[37]	70	70f
64285		GH47	Cand. α-mannosidase		[38]	1421	1421a
55319	abf3	GH54	Cand. α-L-arabinofuranosidase	CBM42	[31]	5126	5126a
123283	abf1	GH54	α-L-arabinofuranosidase I	CBM42	[30]	5126	5126a
121746	gluc78	GH55	Cand. exo-1,3-β-glucanase		[58]	235	235a
54242		GH55	Cand. β-1,3-glucanase		[38]	235	235b
70845		GH55	Cand. β-1,3-glucanase		[38]	235	235b
108776		GH55	Cand. β-1,3-glucanase		[38]	235	235b
56418		GH55	Cand. β-1,3-glucanase		[38]	235	235c
73248		GH55	Cand. exo-1,3-β-glucanase		[38]	235	235d
73643	egl4/cel61a	GH61	Cand. copper-dependent polysaccharide monooxygenase/endo-β-1,4-glucanase	CBM1	[13]	77	77a
120961	cel61b	GH61	Cand. copper-dependent polysaccharide monooxygenase		[16]	77	77b
22129		GH61	Cand. copper-dependent polysaccharide monooxygenase		[38]	515	515a
31447		GH61	Cand. copper-dependent polysaccharide monooxygenase		This study	515	515b
76065		GH61	Cand. copper-dependent polysaccharide monooxygenase		This study	515	515b
27554		GH61	Cand. copper-dependent polysaccharide monooxygenase		[31]	8230	8230a
76210	abf2	GH62	Cand. α-L-arabinofuranosidase		[16]	3103	3103a
22072		GH63	Cand. processing α-glucosidase		[38]	1244	1244a
75036		GH63	Cand. α-glucosidase		[38], A	2428	2428a
65137		GH64	Cand. endo-1,3-β-glucanase		[38]	3917	3917a
123639		GH64	Cand. endo-1,3-β-glucanase		[38]	3917	3917a
124175		GH64	Cand. endo-1,3-β-glucanase		[38]	3917	3917b
25224		GH65	Cand. α,α-trehalase		[38]	2891	2891a
123456		GH65	Cand. α,α-trehalase		[38]	2891	2891a
72526	glr1	GH67	α-Glucuronidase		[29]	5113	5113a
71532		GH71	Cand. α-1,3-glucanase		[37]	287	287a
108672		GH71	Cand. α-1,3-glucanase	CBM24	[38]	287	287b
120873		GH71	Cand. α-1,3-glucanase	CBM24	[38]	287	287b
73179		GH71	Cand. α-1,3-glucanase		[37]	287	287c
22914		GH72	Cand. β-1,3-glucanosyltransferase	CBM43	[38]	111	111a
82633		GH72	Cand. β-1,3-glucanosyltransferase		[37]	111	111a
77942		GH72	Cand. β-1,3-glucanosyltransferase		[38]	111	111b
78713		GH72	Cand. β-1,3-glucanosyltransferase		[47]	111	111c
123538		GH72	Cand. β-1,3-glucanosyltransferase		[38]	111	111d
49081	cel74a	GH74	Xyloglucanase	CBM1	[16]	4769	4769a
42152		GH75	Cand. chitosanase		[38]	3832	3832a
70341		GH75	Cand. chitosanase		[38]	3832	3832a
66789		GH75	Cand. chitosanase		[38]	3832	3832b
49409		GH76	Cand. α-1,6-mannanase		[38]	129	129a
67844		GH76	Cand. α-1,6-mannanase		[38]	129	129a
69123		GH76	Cand. α-1,6-mannanase		[38]	129	129b
122495		GH76	Cand. α-1,6-mannanase		[38]	129	129b
53542		GH76	Cand. α-1,6-mannanase		[38]	129	129c
55802		GH76	Cand. α-1,6-mannanase		[38]	129	129c
74807		GH76	Cand. α-1,6-mannanase		[47]	3765	3765a
27395		GH76	Cand. α-1,6-mannanase		[38]	4345	4345a
58887		GH78	Cand. α-L-rhamnosidase		[38]	3807	3807a
71394		GH79	Cand. β-glucuronidase		[38], A	5190	5190a
73005		GH79	Cand. β-glucuronidase		[38], A	5190	5190a
106575		GH79	Cand. β-glucuronidase		[38], A	5190	5190a
72568		GH79	Cand. β-glucuronidase		[37], A	5190	5190b
73256		GH81	Cand. endo-1,3-β-glucanase		[38]	722	722
79602		GH81	Cand. endo-1,3-β-glucanase		[38]	722	722a
58117		GH89	Cand. α-N-acetylglucosaminidase		[38]	6562	6562a
69700		GH89	Cand. α-N-acetylglucosaminidase		[38]	6562	6562a
74198		GH92	Cand. α-1,2-mannosidase		[38]	318	318a
111733		GH92	Cand. α-1,2-mannosidase		[38]	318	318a
79921		GH92	Cand. α-1,2-mannosidase		[38]	318	318b
60635		GH92	Cand. α-1,2-mannosidase		[38]	318	318c
55733		GH92	Cand. α-1,2-mannosidase		[38]	318	318d
57098		GH92	Cand. α-1,2-mannosidase		[47]	318	318e
69493		GH92	Cand. α-1,2-mannosidase		[38]	318	318f
72488		GH95	Cand. α-L-fucosidase		[38]	2951	2951
5807		GH95	Cand. α-L-fucosidase		[38]	2951	2951a
111138		GH95	Cand. α-L-fucosidase		[37]	2951	2951a
58802		GH95	Cand. α-L-fucosidase		[38]	2951	2951b
4221		GH105	Cand. rhamnogalacturonyl hydrolase		This study	4036	4036a
57179		GH105	Cand. rhamnogalacturonyl hydrolase		[37]	4065	4065a
79606		GH115	Cand. xylan-α-1,2-glucuronidase or α-(4-O-methyl)-glucuronidase		This study	3202	3202a
103033		PL7	Cand. alginate lyase		[38]	9526	9526a
110259		PL7	Cand. alginate lyase		[38]	9526	9526a
111245		PL8	Cand. chondroitin lyase		[38]	10699	10699a
53186	TrGl	PL20	Glucuronan hydrolase		[91]	5536	5536a
69189		PL20	Cand. endo-β-1,4-glucuronan lyase		This study	5536	5536a
108348		GH	-			10159	
105288		GH	-			29033	
121136		GH	-			29033	

Glycoside hydrolase, carbohydrate esterase (excluding CE10) and polysaccharide lyase genes of *T. reesei*, the annotation and functional diversification of the genes

(a), gene identifier as in *T. reesei* v2.0 data base [38]; (b), name given to the gene in the publication/data base marked in the reference column; (c), family and class of the enzyme according to the CAZy classification [5]; (d), cellulose binding module present in the protein; (e), reference to previous studies or to *T. reesei* database versions 1.2 and 2.0.; (f), protein cluster the *T. reesei* protein was assigned to when the protein clusters were mapped to CAZy database by a blast search; (g), functional subgroups within the protein cluster determined according to phylogenetic analysis. A, a previous annotation has been specified/updated during this study.

### Annotation of *T. reesei* CAZyme genes

The annotations of the CAZyme genes of *T. reesei* were specified/updated (the information is summarized in the Additional file 3). The criteria used for the annotation were the relationship of CAZy database members and *T. reesei* CAZymes in the phylogenetic trees, the best blast hits in the CAZy database, predicted functional domains in the proteins ([55,56], Additional file 4), together with the function predictions in the *T. reesei* v2.0 data base [37,38]. The list of the 49 fungi in the protein homology clusters, with their abbreviations are shown in Additional file 5. For selected cases, alignment of the candidate CAZyme against the PFAM profile of the CAZy family was also used to confirm the CAZy family of the candidate gene. By these means, the *T. reesei* CAZyme genes, excluding glycosyltransferase and the family CE10 genes, were concluded to contain 201 GH (glycoside hydrolase) genes, 22 CE (carbohydrate esterase genes) and 5 PL (polysaccharide lyase) genes. The outcome of the annotation process and the functional diversification observed is summarised in Table 1. Phylogenetic trees showing the functional diversifications discussed in more detail, are shown in Additional file 6. In this study we focus on putative CAZymes involved in degradation of plant cell wall derived material, whereas proteins with other functions such as glycosylation or degradation of cell wall components were given less attention. Especially, the function predictions obtained for genes without previous annotation, or genes for which only a general prediction was available, are discussed.

### Cellulase protein homology clusters

A good example to support the functional diversification predictions based on a phylogenetic analysis was the diversification of the well characterised GH7 genes, the cellobiohydrolase *cbh1* and endoglucanase *egl1*. The encoded proteins are assigned to the same protein homology cluster but different functional subgroups in accordance with the known enzymatic activities of the proteins (Table 1A, Additional file 6). The third major component of the cellulolytic system, cellobiohydrolase CBHII belongs to the family GH6 and is the only member of the family in *T. reesei*. Endoglucanases can be found also in the glycoside hydrolase families GH5, GH12 and GH45. GH5 includes the characterised EGII, a candidate membrane-bound endoglucanase CEL5B in the same homology cluster and functional subgroup, and an additional endoglucanase candidate (53731) in a separate cluster (Table 1A). The other GH5 members include the β-mannanase MANI, and a candidate for a β-1,3-mannanase/endo-β-1,4-mannosidase (71554), glucan β-1,3-glucosidase (64375), endo-β-1,6-glucanase (64906) and a β-glycosidase (77506) without a specific functional prediction. GH12 family includes the characterised endoglucanase EGIII together with a candidate endoglucanase (77284) but in separate protein homology clusters (Table 1A). The endoglucanase EGV of the family GH45, as well as the xyloglucanase CEL74A of the family 74, are the only members of their families in *T. reesei*. GH61 family member EGIV/CEL61A has previously been described as an endoglucanase [13]. However, recently the family has been suggested to act in the degradation of lignocellulose material via an oxidative mechanism [18]. Based on our analysis, *T. reesei* genome harbours five candidate GH61 members in addition to EGIV/CEL61A. The encoded proteins are divided into three protein homology clusters and four functional subgroups inside the clusters (Table 1C).

**Figure 1:**
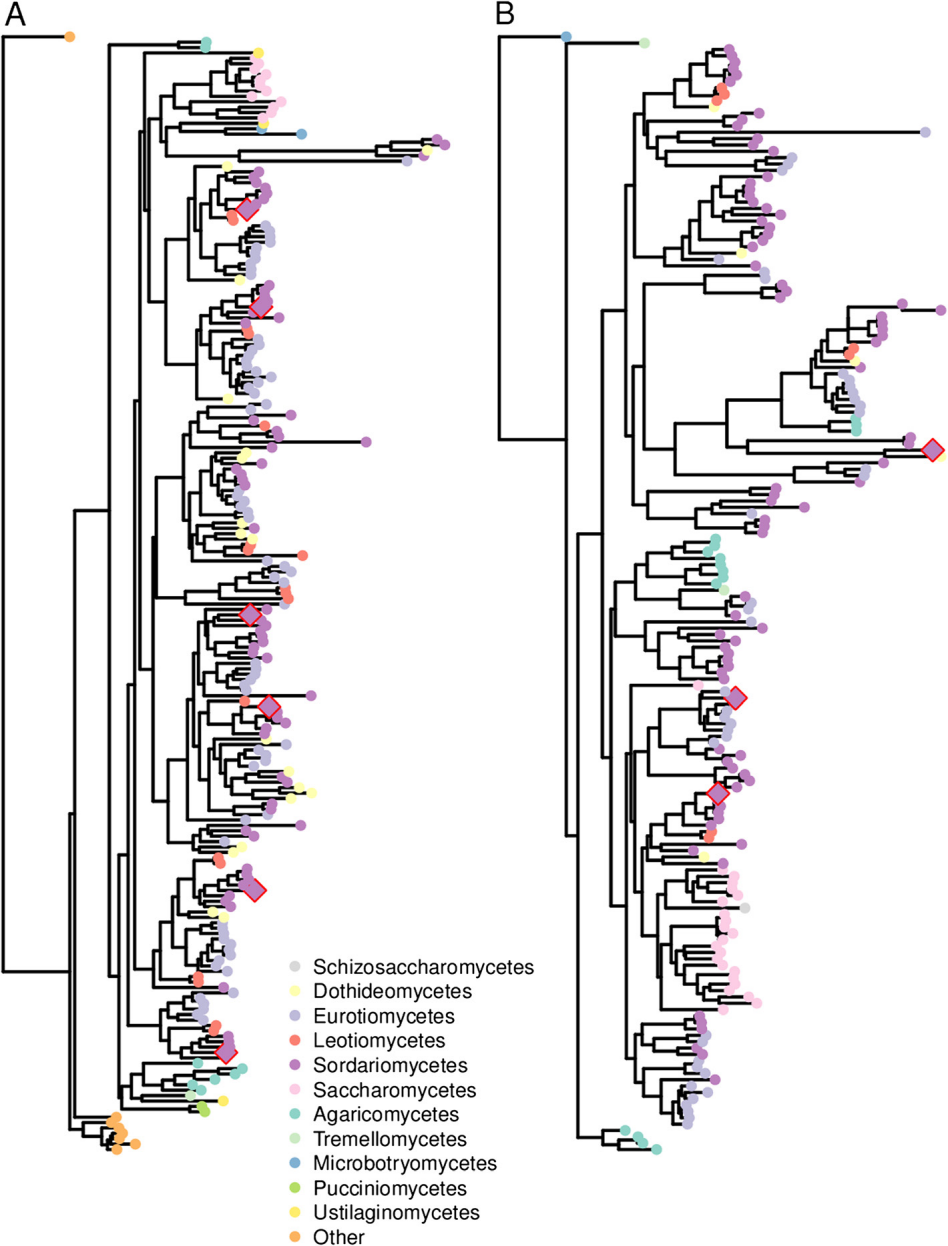
**Phylogeny of fungal β-glucosidases of family GH3.** Phylograms containing the *T. reesei* proteins and homologous proteins of 48 other fungi in the database were constructed. (**A**), protein cluster 110; (**B**), protein cluster 132. Classification of the fungi is indicated with couloured symbols, *T. reesei* proteins marked with a diamond bordered with red. For a detailed presentation of the tree, including the abbreviations for the fungal strains and the Uniprot identifiers, see Additional file 6.

In the case of β-glucosidases, experimental evidence supporting functional differences has only been obtained for BGLI and BGLII, the first being the major extracellular β-glucosidase and the second being an intracellular enzyme. However, phylogenetic analysis suggests that further functional differences may exist within this group of enzymes (Table 1A). Altogether, the *T. reesei* genome encodes eleven characterised or predicted β-glucosidases, two belonging to the family GH1 and nine to the family GH3. The intracellular GH1 β-glucosidases of *T. reesei* (BGLII and CEL1B, the latter predicted to be intracellular due to the lack of predicted signal sequence) are in the same protein homology cluster but in different functional subgroups. The β-glucosidases of family GH3 are divided into two homology clusters, and furthermore, showed diversification within the clusters, so that the genes could be assigned to nine groups. Functional diversification of the GH3 β-glucosidases within the same protein homology cluster is visualised in Figure 1 and Additional file 6. The predicted β-glucosidases 47268, 66832 and 104797 are assigned to the same cluster as BGLI, CEL3B and CEL3E, and 108671 to the same cluster as CEL3C and CEL3D. The GH3 β-glucosidases BGLI, CEL3E, CEL3B, 66832, 104797 and 108671 are predicted to be secreted according to the signal sequence prediction (SignalP 4.0, [57]).

### Hemicellulase and other CAZyme protein homology clusters

In addition to β-glucosidases, the family GH3 includes candidate β-N-acetylglucosaminidases (69557 and 79669) and β-xylosidases (BXLI and candidate 58450). The β-N-acetylglucosaminidases and β-xylosidases are found in separate protein homology clusters according to their (predicted) functions, and the β-xylosidases are also in separate functional subgroups (Table 1A). In addition to the GH3 β-xylosidases, *T. reesei* is predicted to encode a candidate β-xylosidase (73102) of the family GH39, and two proteins of the family GH43 predicted to have either β-xylosidase or α-L-arabinofuranosidase activity (68064, 3739), all in separate protein homology clusters (Table 1B). The characterised arabinofuranosidase ABFI and the candidate enzyme ABFIII of the family GH54 are not functionally diversed from each other according to the functional diversification criteria (Table 1C). In addition to these, the *T. reesei* genome also encodes a candidate arabinofuranosidase ABFII of the family GH62.

*T. reesei* has four characterized xylanases and two candidate xylanases. The characterized xylanases belong to families GH10 (XYNIII), GH11 (XYNI*,* XYNII) and GH30 (XYNIV). Candidate xylanases can be found from families GH11 (112392) and GH30 (69276). The candidate xylanase 112392 (also called XYNV in a recent publication, [58]) is assigned to the same protein homology cluster as XYNI and XYNII and is in the same functional subgroup as XYNI (Table 1A). Candidate xylanase 69276 is in the same homology cluster and functional subgroup as XYNIV (Table 1B).

The carbohydrate esterase families CE3 and CE5 contain known and/or candidate acetyl xylan esterases of *T. reesei*. The carbohydrate esterase family 5 includes both acetyl xylan esterases and cutinases (Table 1A). The candidate acetyl xylan esterases (54219 and AXEII) are in the same protein homology cluster with the characterized enzyme AXEI, the 54219 belonging to the same functional subgroup as AXEI. The candidate cutinase (60489) clusters together with known and candidate cutinase homologues of other fungi. Our study also revealed candidate members of CE3 family, including candidates for acetyl xylan esterases or esterase/suberinase. However, the ORF prediction (in the genome database [38]) for majority of the genes is unclear due to difficulties in prediction of N- or C-termini or intron positions, which hampers phylogenetic analysis of the genes.

Hemicellulases of *T. reesei* are also present in families GH2 and GH27. The family GH2 is one of the families including members with versatile enzymatic activities. The predicted members of GH2 of *T. reesei* include five candidate β-mannosidases (5836, 69245, 59689, 57857 and 62166) belonging to the same protein homology cluster but to three different functional subgroups within the cluster (Table 1A). GH2 members include also a candidate exo-β-D-glucosaminidase (77299), and a candidate enzyme with a predicted function as a β-galactosidase or β-glucuronidase (76852). The six candidate α-galactosidases (27219, 27259, 59391, 75015, 55999 and 65986) of family GH27 are divided to two protein homology clusters (Table 1B). Proteins encoded by genes 27219, 27259, 59391 and 75015 are assigned to the same cluster as AGLIII and are not functionally diversified either from AGLIII or from each other (cluster contained only *T. reesei* proteins). The remaining candidate α-galactosidases are in the same cluster as AGLI and are divided to two functional subgroups within the cluster.

Our study also suggested a new member of CE16 family and of PL20 family belonging to the same functional subgroup as the characterised AESI and TRGL, respectively (Table 1A and 1C). In addition to the known GH67 α-glucuronidase (GLRI), a GH115 type of α-glucuronidase (α-1,2- or α-(4-O-methyl)-glucuronidase) was predicted (79606). The *T. reesei* genome was also found to encode four candidate GH79 β-glucuronidases (71394, 106575, 72568, 73005) not identified previously, but which are probably involved in proteoglycan hydrolysis rather than lignocellulose degradation. Also an additional member of GH105 family, a predicted rhamnogalacturonyl hydrolase (4221), was identified in the study in addition to the previously predicted one (57179).

### Comparison of *T. reesei* CAZyme homology clusters with other fungi

Comparison of *T. reesei* protein homology clusters with other fungi by looking at the number of genes per species in the clusters, revealed several interesting differences (Additional file 7). The cluster containing AGLIII and four candidate α-galactosidases is unique to *T. reesei*. This protein homology cluster is not found from any other of the 48 fungi included in this study. The cluster containing four candidate β-glucuronidases from family GH79 is expanded in *T. reesei* as compared to other fungi. *T. reesei* has 4 genes encoding these proteins while the other 48 fungi in this study contained only 0 to 3 genes. Similarly, the cluster containing candidate extracellular or membrane bound chitinases is expanded to include 6 genes in total, while in most of the 48 fungi there are 0 to 5 genes. This cluster corresponds to phylogenetic group B as described in a previous publication on chitinase phylogeny [59]. The cluster also contains chitinase CHI18-18 which was not included in group B. Expansion of GH18 genes of *T. reesei* has been described previously and it has been suggested to be involved in functions related to pathogenicity to other fungi [37].

The cluster containing seven genes from family GH92 is not found in *Fusarium* species that are the closest relatives of *Trichoderma* in our data set. It is possible that *Fusarium* genes have diversified further apart while *Trichoderma* genes have retained the ancestral functions. One of the two clusters that contain genes encoding members of family GH43 is hugely reduced in *T. reesei* compared to other Pezizomycotina species, especially *Fusarium* spp. *T. reesei* has only one gene in this cluster while *Fusarium oxysporum* has 12. Reduction is also visible in two protein homology clusters containing members from the family GH61. The reduction is especially notable in the cluster number 77 where *T. reesei* has only two genes while the number of genes in other Pezizomycotina can be as high as 43. These two reductions were already noticed during the initial genome analysis of *T. reesei*[37].

### Horizontal gene transfer

Our study revealed also several cases of putative horizontal gene transfer from bacteria. As mentioned above, proteins encoded by genes 59791 and 73101 were assigned to protein homology clusters with no other fungal proteins i.e. they had no significant homologues in the fungal genomes included in the clustering. However, the proteins had homologues in the closely related *Hypocrea* (*Trichoderma*) species as well as a large number of bacterial homologues. 73101 had several bacterial GH16 family proteins as homologues, and a candidate endo-β-1,3-1,4-glucanase of *Bacillus subtilis* as the best blast hit in the CAZy database. 59791was closely related to GH18 family chitinases of *Hypocrea* (*Trichoderma*) species, and had homologues also especially in *Streptomyces* and *Bacillus* species*.* The phylogeny of the genes thus suggests possible gene transfer from bacteria. The possibility of horizontal gene transfer of 59791 has also been discussed in a previous publication [59]. In addition, a candidate β-glucosidase (108671) of GH3 was shown to be possibly a result of horizontal gene transfer. Although the protein is assigned to the same homology cluster as CEL3D and CEL3C beside proteins from other *Trichoderma* species, the closest homologous for this protein are from bacteria. The phylogenetic analysis of these genes are represented in additional files (59791 in Additional file 8, 73101 in Additional file 9, and 108671 in Additional file 10).

### Identification of similarly expressed genes by visualization of the expression data

The induction of *T. reesei* CAZyme genes was studied by cultivating the fungus in the presence of different inducing substrates and analysing the transcriptional responses at different time points of induction using oligonucleotide microarrays. The transcript signals in induced cultures were compared to the ones in uninduced control cultures at the same time point. The microarray expression data on the CAZyme genes is represented in detail in the Additional file 11 (including the normalised log2 scale signal intensity of the genes in each condition, the fold change of the signal in the induced culture as compared to the uninduced control cultures at the same time point, and statistical significance of the difference in expression as compared to the control cultures at a corresponding time point). The substrates used in the study were differentially pretreated bagasse materials (ground bagasse without further pretreatment, bagasse pretreated by steam explosion, and bagasse hydrolysed enzymatically after steam explosion pretreatment), pretreated wheat straw and pretreated spruce, birch xylan, oat spelt xylan, Avicel cellulose and sophorose. Based on liquid chromatographic analysis of the carbohydrate content of the pretreated complex substrates (Table 2) and information obtained from the manufacturers of the other substrates used, bagasse and wheat straw are the most complex ones. Steam exploded wheat straw and pretreated bagasse material contain cellulose and arabinoxylan, but also polysaccharides with other substitutions, such as galactose or mannose units, whereas pretreated spruce consists mostly of cellulose. Sophorose is a disaccharide, 2-O-β-D-Glucopyranosyl-α-D-Glucose, that can be produced via transglycosylation reaction from cellobiose, a cleavage product of cellulose.

**Table 2:** Carbohydrate composition of the pre-treated biomass substrates

**Sample**	**Rhamnose**	**Arabinose**	**Galactose**	**Glucose**	**Xylose**	**Mannose**	**Fructose**
BS	< 0.1	0.54	0.15	59.5	12.59	0.24	< 0.1
BE	< 0.1	0.62	0.14	33	9.68	0.38	< 0.1
WH	< 0.1	0.38	< 0.1	61.6	2.96	0.12	-
SP	< 0.1	< 0.1	< 0.1	46	< 0.1	< 0.1	< 0.1

The amounts of the different carbohydrates are shown as mg/100 mg or dry matter. BS, steam exploded bagasse; BE, enzymatically hydrolysed bagasse; WH, wheat straw; SP, spruce.

**Figure 2:**
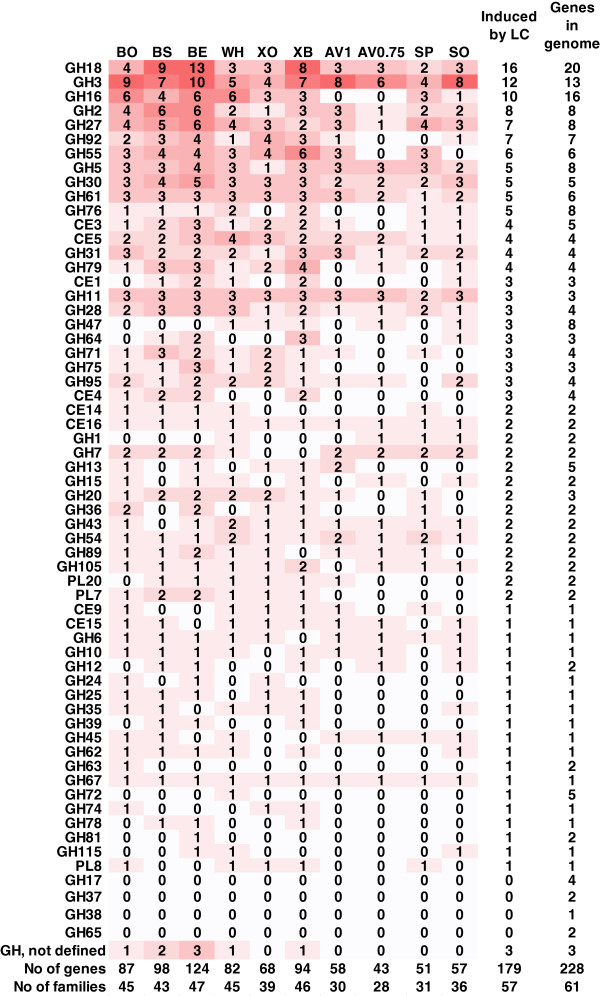
**CAZy family members induced in the presence of the different lignocellulose substrates.** The number of genes in each CAZy family induced in the presence of the different lignocellulose substrates: BO, ground bagasse; BS, steam exploded bagasse; BE, enzymatically hydrolysed steam exploded bagasse; XO, oat spelt xylan; XB, birch xylan; AV1, 1% Avicel cellulose; AV0.75, 0.75% Avicel cellulose; WH, steam exploded wheat straw; SP, steam exploded spruce; SO, sophorose. The intensity of the colour represents the amount of genes induced. The total number of genes in each CAZy family induced by at least one of the substrates, and the total number of the CAZy family members in *T. reesei* genome are shown on the right. The total number of genes induced by the substrate and the number of CAZy families the genes belong to, are shown at the bottom. LC, lignocellulose substrate.

The majority (179 genes) of the 228 CAZyme genes (excluding GT and CE10 family genes) were induced by at least one of the substrates used (higher expression level in the induced cultures as compared to the uninduced cultures at the same time point). The largest number of genes and CAZy families induced was detected in the cultures with the hemicellulosic material, bagasse, xylans and wheat straw (68–124 genes in 39–47 CAZy families), whereas cultivation in the presence of cellulosic or cellulose derived materials, Avicel cellulose, pretreated spruce, or sophorose, resulted in a clearly smaller number of genes induced (43–58 genes in 28–36 families). The number of induced genes within each CAZy family is represented in Figure 2.

Based on the microarray data, the common core of genes induced in the presence of the lignocellulose substrates (as judged by induction of gene expression by both cellulose and xylan, and induction in the presence of at least 70% of the substrates used) included genes encoding characterised or predicted functions as GH6 cellobiohydrolase, GH5 endoglucanase, xylanases of families GH10, GH11 and GH30, GH5 β-mannanase, GH3 family β-glucosidases and β-xylosidases, GH27 α-galactosidases, GH2 β-mannosidases, acetyl xylan esterases of families CE3 and CE5, glucuronoyl and acetyl esterases of families CE15 and CE16, GH31 α-glucosidases/α-xylosidases, GH54 and GH43 α-L-arabinofuranosidases (or β-xylosidase/α-L-arabinofuranosidases), GH61 polysaccharide monooxygenases, GH55 β-1,3-glucanases, GH67 α-glucuronidase, GH79 β-glucuronidase, GH105 rhamnogalacturonyl hydrolase, GH95 α-L-fucosidase, GH89 α-N-acetylglucosaminidase, and chitinases of families GH18 and GH20.

**Figure 3:**
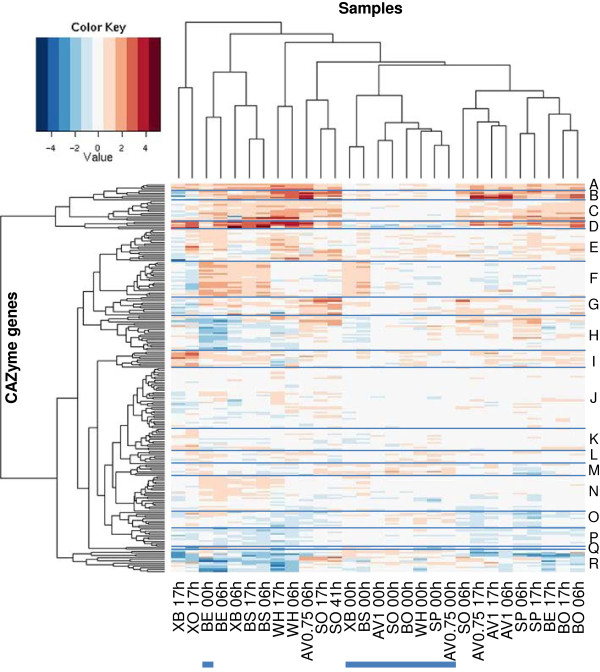
**Heat map representing expression profiles of *T. reesei *CAZyme genes when the fungus was grown on different lignocellulose substrates.** The fold change of the expression signal in the induced culture vs. the signal in the uninduced cultures at the same time point for each gene is represented by the color code (see the color key for the log2 scale fold changes). The data for each gene is represented as rows (for gene IDs from top to bottom see the Additional file 11, column a), and the samples collected at different time points of induction (0 h, 6 h, or 17 h) in the presence of the different substrates are shown as columns. BO, ground bagasse; BS, steam exploded bagasse; BE, enzymatically hydrolysed steam exploded bagasse; XO, oat spelt xylan; XB, birch xylan; AV1, 1% Avicel cellulose; AV0.75, 0.75% Avicel cellulose; WH, steam exploded wheat straw; SP, steam exploded spruce; SO, sophorose. The heat map was divided to 18 sub-branches (A-R) according to similarities in expression profiles. The zero time points are indicated by a blue line at the bottom.

The analysis revealed also a more refined pattern of co-expressed genes. To visualise the co-expressed gene groups as well as differences in the induction pattern of the genes in the presence of different substrates, a heatmap representation was used (Figure 3). The heatmap was generated using the fold changes of the transcript signals in the induced cultures vs. the uninduced cultures at the same time point for each of the genes.

In the heatmap, the branches A, B, C and D represent genes that are induced by the presence of most of the substrates, and include many of the known cellulolytic and hemicellulolytic genes. Branch A contains genes that are rather evenly induced by all the substrates (*egl4/cel61a*, *bxl1*, *xyn1*, *xyn4*). The genes give strong signals in the inducing conditions, and have moderately high basal signal levels also in the uninduced conditions. The genes in branch B have an especially pronounced induction by the presence of Avicel cellulose and wheat, but reduced expression at the late time points of xylan cultures (candidate GH28 exo-polygalacturonase *pgx1*, candidate GH3 β-glucosidase *cel3d*, GH5 β-mannanase *man1*, candidate GH2 β-mannosidase, candidate GH61 polysaccharide monooxygenase *cel61b*). The genes in branch C are moderately induced by most of the substrates, but similarly to the branch B, many of the genes show reduction of the signal at the late time points of xylan cultures (candidate GH3 β-glucosidase *bgl3f*, candidate CE3 acetyl xylan esterase 41248, candidate GH18 chitinase *chi18-7*, CE16 acetyl esterase *aes1*, candidate GH105 rhamnogalacturonyl hydrolase 57179, candidate GH95 α-L-fucosidase 5807, GH27 α-galactosidase *agl3*, candidate GH55 β-1,3-glucanases 56418 and 54242). The branch D represents genes induced by most of the substrates but especially by the presence of xylans, steam exploded bagasse and wheat straw. In accordance with the strong induction by xylans, the branch D includes both known and candidate hemicellulase genes (GH67 α-glucuronidase *glr1*, GH11 endo-β-1,4-xylanase *xyn2*, candidate GH11 endo-β-1,4-xylanase *xyn5*, CE5 acetyl xylan esterase *axe1* and a candidate CE5 acetyl xylan esterase).

Other distinctive groups of co-regulated genes are induced by subset of the substrates studied. The branch F is characterised by genes giving rather low or moderate signals in the array analysis and showing induction by birch xylan, steam exploded and enzymatically treated bagasse already at the early time points. This group includes e.g. candidates of GH16, GH18, GH27 and GH92 family genes among others. The genes in branch E show moderate expression and induction levels, mostly in the presence of wheat straw, but subgroups of the branch also with bagasse, spruce, oat spelt xylan or sophorose. The branch G represents genes induced especially by sophorose. This group contains many genes known to encode enzymes with activity on lignocellulose substrates, (GH3 β-glucosidase *bgl1/cel3a*, a candidate GH3 β-glucosidase *cel3e*, GH7 endo-β-1,4-glucanase *egl1/cel7b*, GH10 endo-β-1,4-xylanase *xyn3*, GH74 xyloglucanase *cel74a*, CE15 glucuronoyl esterase *cip2*) as well as candidates for GH30 endo-β-1,4-xylanase, and GH79 β-glucuronidase among others. Branch H contain genes that have moderately high signal levels and are induced in the presence of the cellulosic substrates, spruce, sophorose and Avicel, but whose signal levels are reduced in the presence of pretreated bagasse, wheat straw and birch xylan. This group contains especially candidate α-1,2-mannosidase genes of the family GH47, but also genes of families GH3 (β-glucosidase *cel3c)*, GH5, GH16, GH17, GH27, GH55, GH76, and GH95. The branch I contains genes with the strongest induction at the late time point of xylan cultures (a candidate GH3 β-xylosidase *xyl3b*, a candidate GH3 β-glucosidase *cel3b*, GH36 α-galactosidase *agl2*, a candidate GH79 β-glucuronidase, a candidate PL7 polysaccharide lyase, chitinase genes of families GH18 and GH20 and a candidate GH31 α-glucosidase among others). The branch J contains genes with rather strong constitutive signals. Among others, the group contains both GH1 family β-glucosidase genes, a candidate GH3 family β-glucosidase gene, two GH5 family genes (*cel5b* and *cel5d*), a candidate CE5 acetyl xylan esterase *axe2*, a candidate CE3 family esterase, a candidate GH43 family β-xylosidase/arabinofuranosidase and a novel candidate GH115 family gene. For detailed gene content of the branches, see the Additional file 11.

The major cellobiohydrolase genes, *cbh1* and *cbh2*, are erroneously included to branch J due to saturated signal level in the microarray analysis. The expression of these genes was studied separately using q-PCR (see below).

The microarray study also revealed a group of genes whose expression level was increased immediately after addition of the substrate, but induction (as compared to the control cultures) ceased soon after that. These genes can be found especially in heatmap branches I, L, M, O, and R (Figure 3). These included e.g. genes encoding two candidate β-mannosidases (5836 and 69245), candidate β-xylosidase (58450), a chitinase (*chi18-3*), two α-L-arabinofuranosidases (*abf1* and *abf3*), two candidate α-1,2-mannosidases (74198 and 60635), a candidate endo-polygalacturonase (103049), endoglucanase (*egl3*), a candidate GH2 family protein (102909), two α-galactosidases (*agl1* and *agl2*) and a glycoside hydrolase for which a family could not be assigned (105288). From these genes *chi18-3*, *egl3*, *abf3* and genes 74198, 5836, 69245, 102909, 105288 and 60635 were induced at the early time point by at least five of the substrates. It is notable that several genes induced at early time point encode enzymes that are involved in hemicellulose degradation (mannose backbone degradation, releasing side chains from hemicellulose and digesting oligosaccharides derived from hemicellulose).

**Figure 4:**
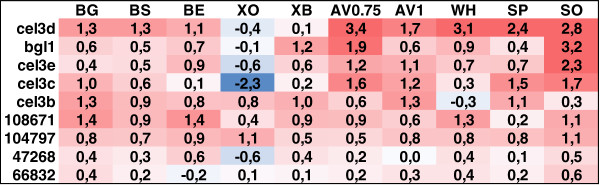
**Expression profiles of *T. reesei *GH3 β-glucosidase genes.** The maximal induction level in each of the culture conditions is shown as the fold change of the signal in the induced cultures vs. the signal in the uninduced cultures at the time point when the induction was the highest (log2 scale). The blue and red colours represent negative and positive changes in the expression, respectively. The intensity of the colour is proportional to the magnitude of induction/repression. BO, ground bagasse; BS, steam exploded bagasse; BE, enzymatically hydrolysed steam exploded bagasse; XO, oat spelt xylan; XB, birch xylan; AV1, 1% Avicel cellulose; AV0.75, 0.75% Avicel cellulose; WH, steam exploded wheat straw; SP, steam exploded spruce; SO, sophorose.

Due to the importance of β-glucosidases in the total hydrolysis of lignocellulose biomass and the observation that members of GH3 are abundantly induced by different substrates and are functionally very diverse according to the phylogenetic analysis, the induction of GH3 β-glucosidase genes was inspected in more detail (Figure 4). The expression patterns of the GH3 β-glucosidase genes differed clearly from one another. A set of the genes was induced most strongly in the presence of sophorose and Avicel cellulose, but not that much on the xylans, whereas some of the genes showed equal or higher induction in the presence of bagasse and xylans as in the presence of the cellulosic materials, and some lacked induction by the substrates. In addition, the induction pattern of the GH3 β-glucosidase genes showed gene specific features. Gene *cel3d* showed strong induction by all the substrates except for the xylans. The strongest induction was obtained in the presence of Avicel, wheat, spruce and sophorose. Genes *bgl1/cel3a* and *cel3e* were strongly induced on sophorose, but also showed a milder induction by many other substrates, especially in the presence of Avicel. Gene *cel3c* was induced by sophorose and Avicel, but also by spruce and ground bagasse. Genes *cel3b* and 108671 were moderately induced by the majority of the substrates except for *cel3b* on wheat and sophorose and 108671 on oat spelt xylan and spruce. Gene 104797 was mildly induced by all the substrates, most by oat spelt xylan and sophorose. Genes 47268 and 66832 were hardly at all induced by the substrates studied.

47 CAZyme genes did not show up-regulation in the presence of any of the substrates as compared to the control cultures (Additional file 11). Most of the uninduced genes are predicted to encode functions other than lignocellulose degradation. Especially, the uninduced genes included genes encoding proteins likely to be involved in processing of cell wall components, chitin processing/degradation, utilisation of storage carbon or 1,4-α-glucan substrates, or protein glycosylation. Expression information could not be obtained for four genes that are absent from the Rut-C30 genome due to a large deletion in scaffold 15 (the genes 25224, 64906, 65215 and 122780) [60,61].

### Identification of expression of highly induced genes and confirmation of microarray results by quantitative PCR

**Figure 5:**
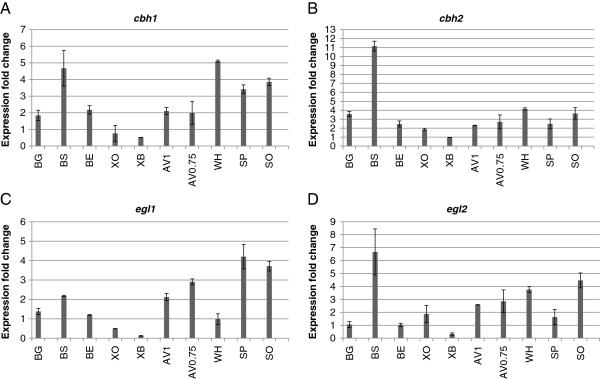
**Quantitative PCR analysis of highly expressed genes.** Samples collected at 17 h time point of induction were subjected to the qPCR analysis. The expression levels normalised with *gpd1* signal are shown in the graphs as a fold change as compared to the uninduced control cultures at the same time point. The values are means of two biological replicates, and the error bars indicate standard deviation. (**A**) Expression of *cbh1*, cellobiohydrolase 1; (**B**) expression of *cbh2*, cellobiohydrolase 2; (**C**) expression of *egl1*, endoglucanase 1; (**D**) expression of *egl2*, endoglucanase 2.

The major cellulase genes, *cbh1* and *cbh2,* are among the most abundantly expressed genes in *T. reesei* (within the top 0.5% of the genes in the dataset)*.* In the microarray analysis, the induction of the genes was barely detectable due to saturation of the signal levels. It was also suspected that the magnitude of induction of the major endoglucanase genes, *egl1* and *egl2,* might be too low in the microarray analysis. In order to study the expression of these genes, a quantitative PCR analysis of samples collected at the 17 hour time point of induction was carried out. According to the qPCR analysis, *cbh1* and *cbh2* were induced in the presence of all other substrates except for *cbh2* on birch xylan, and *cbh1* on neither of the xylans (Figure 5). Endoglucanase gene *egl1* was induced in the presence of ground and steam exploded bagasse, Avicel, spruce and sophorose and *egl2* was induced especially in the presence of steam exploded bagasse, but also in the presence of Avicel, wheat, spruce, sophorose and oat spelt xylan. Furthermore, the array data on the earlier time points of induction showed induction of *egl2* also on ground and enzymatically pretreated bagasse as well as on birch xylan.

In addition, a set of genes covering both abundantly and moderately expressed genes were included in the qPCR analysis in order to validate the microarray method and to investigate the detection limits of the two methods. The additional set of genes in the qPCR analysis included *abf1, axe1, bxl1, cel3c, cel3d, cel61b*, *swo1, xyn1, xyn2, xyn3* and *xyn4.* A scatter plot comparing the log2 signal intensities of the microarray data to the Cp values of qPCR data is represented in the Additional file 12. The log2 signal intensities of the microarray data correlate reasonably well with qPCR data at microarray signal level below 15, above which the microarray signal start to become saturated.

## Discussion

Although *T. reesei* is an important producer of enzymes for industry and biorefinery applications, little is known about the expression of the enzyme genes in the presence of complex biomass substrates. In this study, the expression of the CAZyme genes of *T. reesei* was studied using several substrates as inducers of gene expression. Substrates included complex biomass materials that are of interest from a biorefinery point of view, as well as purified polysaccharides and a simple inducing disaccharide. In addition, the annotations and functional predictions of *T. reesei* CAZymes were updated using computational and manual methods, including phylogenetic information. This was done in order to assist deeper understanding of *T. reesei* plant biomass degrading enzymes, their regulation and identification of essential enzymatic activities and enzyme genes for complete biomass degradation. After the initial annotation of the *T. reesei* genome [37], attempts to identify all *T. reesei* cellulolytic and hemicellulolytic genes has been done using genome version 1.2 [62] but to our knowledge this is the first publication after the initial annotation in which the *T. reesei* genome v2.0 has been searched for the CAZyme genes and the phylogenetic data has been thoroughly explored to assist the annotation process.

A BLAST based method together with phylogenetic information was used to identify 201 glycoside hydrolase genes, 22 carbohydrate esterase genes (CE10 genes were left out) and 5 polysaccharide lyase genes in total. Detected discrepancies between the annotations of the genome versions 1.2 [47] and 2.0 [38] and published literature were corrected, and additional refined functional predictions were made for a set of CAZymes based on the analyses. In total 13 putatively new *T. reesei* CAZyme genes were identified during this study (Table 1). Several of these genes belonged to the carbohydrate esterase class which indicates that this group of *T. reesei* enzymes is still less studied than the glycoside hydrolases. Two additional candidate GH61 genes were found emphasizing the possible importance of GH61 enzymes as accessory enzymes in cellulose degradation, although the number of GH61 genes of *T. reesei* is still reduced as compared to other fungi ([37], Additional file 7). For 31 *T. reesei* CAZyme genes the annotation was either refined or a new annotation was given (Table 1). Updated annotations were abundant especially in families GH16, GH17 and GH79. Updated annotations revealed, among others, a fifth candidate GH2 β-mannosidase, a putative ninth GH3 β-glucosidase, a putative second GH12 endoglucanase and the first candidate GH39 β-xylosidase.

In the annotation process, protein homology clusters from 49 fungal species (including *T. reesei*) were mapped to the CAZy database for CAZy family assignment and functional prediction of the genes/gene products, and phylograms of the homology clusters were constructed to assist the annotation of *T. reesei* CAZymes. The phylogenetic relationship of the genes/proteins within the clusters was used to predict further functional diversification of the genes.

Several known and candidate lignocellulose degrading enzymes of *T. reesei* displayed functional diversification within the protein homology clusters, even in the cases in which the enzymes belonged to the same CAZy family and for whom similar activity was predicted based on the closest homologues. Particularly *T. reesei* β-glucosidases of family GH3 and α-galactosidases of GH27 were functionally diverse (Table 1, Figure 1, Additional file 6). It is also worth noting, that GH18 chitinases were extremely diverse by dividing in to as many as five different protein homogy clusters and into 12–13 functional subgroups (Table 1, Additional file 6). A group of chitinases was induced by the lignocellulose substrates used in the study. It is possible that some of the genes encode functions other than merely chitin degradation, since most of the *T. reesei* chitinases are not biochemically characterized. It could also be hypothesized that the saprophytic way of life and pathogenicity towards other fungi would share common regulation mechanisms.

In most cases where the phylogenetic analysis suggested functional diversification, the expression of the genes on different substrates differed as well, as judged based on clustering of the expression profiles (Table 1, Figure 3). A good example of this is the family GH3 β-glucosidases, which were all divided in separate functional subgroups, and whose expression patterns differed from each other. Tight co-regulation was relatively rare among the genes that belonged to the same functional subgroup, also indicating diverted regulation of these genes. However, in a few cases genes of the same functional subgroup were co-regulated. An example of such a case is the GH2 candidate β-mannosidases (5836 and 69245), belonging to the same functional subgroup and showing co-induction immediately after addition of the substrates. The observation that functional diversification is rather common for the CAZymes of *T. reesei* and that the diversification can be seen in differential expression, suggests that the diversified enzymes might be involved in substrate specific processes and/or have different biochemical properties.

The expression analysis of the CAZyme genes in the presence of different substrates revealed distinct groups of co-expressed genes (Figure 3). Part of the CAZyme genes were induced in the presence of both xylan and cellulose type of substrates. The group of genes showing the most consistent induction in the presence of all the substrates used in the experiment contained many genes related to xylanolytic activities. The majority of the CAZyme genes showed differential expression patterns on different types of substrates. Some of the genes exhibited induction especially on the xylan containing material, either on the pure xylans or the complex material containing xylan. The induction of some of the genes was dependent on the type of xylan used in the experiment, suggesting that side chains on xylan may play a role in the induction process. The different types of side chains may contribute also to induction of the genes by different biomass material. The induction of the genes in the xylan cultures also showed temporal differences, some of the genes were induced at the late time points of the induction experiment and some were specific to early stages in xylan cultures or cultures with the xylan containing complex material. A group of genes was induced especially on the cellulosic material, either Avicel or pretreated spruce, and on other complex material to different extent. Part of the cellulose induced genes was induced also in the presence of sophorose. Sophorose can be generated as a transglycosylation product from cellulose degradation product, cellobiose, and therefore could act as a primary inducer in cellulose cultures. Interestingly, a set of genes were induced prímarily by sophorose and only to a lesser extent by the more complex materials. Furthermore, a number of examples were detected where the genes showed stronger induction in the presence of the complex material as compared to the purified polymers, or where the genes were only induced in the presence of the complex material.

Our data also showed a group of CAZyme genes that were induced at the early time points immediately after addition of the inducing substrates, after which their expression declined. Induction only at early time points followed by a decline at later time points could indicate that the enzyme is either required to initialise hydrolysis of the substrate or to be involved in recognizing the polymer substrate and cutting inducing monomers from the substrate.

The results suggest that several regulatory mechanisms, depending on the inducers present, may act on the CAZyme gene promoters simultaneously, and in some cases also in an additive manner. The complex material may also provide other inducing components than the xylan and cellulose derived inducers. Complete hydrolysis of complex biomass derived material most likely requires action of the enzymes as a cascade. At the initial stages certain components are exposed to act as inducers or as sources for inducer formation and certain linkages are accessible for the enzymatic cleavage. As the degradation proceeds, additional components and linkages are exposed requiring other enzymatic activities for cleavage and, different induction mechanisms to produce the enzymes. The regulation of genes encoding xylanolytic enzymes of the model organism *Neurospora crassa* has been suggested to involve several regulatory groups. Xylanase regulator XLR-1 was suggested to work alone or in combination with other unknown regulators and a XLR-1 independent group of genes was also suggested to exist [63]. The results of our study support the theory of several different regulatory groups which may be partly overlapping.

Comparison of transcriptional profiling data sets reveals the partly different regulatory mechanisms employd by different fungi. The most notable differences between the Avicel regulons of *T. reesei* and *N. crassa* are the larger number of *T. reesei* GH3 genes induced as compared to *N. crassa* and the larger amount of *N. crassa* CE1 and GH61 genes induced as compared to *T. reesei*. There are also differences between the xylan induced genes of the two fungi which are partly due to the fact that in contrast to *T. reesei*, *N. crassa* cellulase genes are not induced by xylan [63]. *N. crassa* cellulase genes are also not induced by sophorose [64].

## Conclusions

Computational and manual approaches, also including phylogenetic analysis, was used to update and refine annotation of the CAZyme gene content of *T. reesei* and to study the functional diversification of *T. reesei* CAZyme genes. As an outcome of this study several putatively new CAZyme genes of *T. reesei* were detected, discrepancies between the annotations of the different genome versions and published literature were corrected, and additional refined functional predictions were made for a set of CAZymes. In addition, phylogenetic analysis revealed functional diversification within the CAZy families and enzyme activity groups.

The analysis of *T. reesei* CAZyme gene expression in the presence of different licgnocellulose materials showed a complex pattern of co-regulated groups of genes. Both substrate dependent and temporal differences in the induction of the different groups of genes were detected. The results suggest that several regulatory mechanisms, depending on the inducers present, may act on the CAZyme gene promoters simultaneously, and in some cases the different mechanisms may also act in an additive manner. The complex regulatory system may be required to accomplish complete hydrolysis of biomass derived material by the enzymes produced. Different sets of enzymes are likely to be required to hydrolyse different materials at the different stages of the hydrolysis, thus setting a demand for complex regulatory mechanisms to ensure energetically cost-effective enzyme production in the cells.

Identification of the CAZyme content of *T. reesei* genome together with the expression analysis in the presence of different lignocellulose materials has given evidence for the importance of several yet uncharacterized enzymes in the degradation of biomass substrates and also new information on the enzymes needed for the complete degradation of different lignocellulose substrates. Furthermore, the information on the co-regulated groups of genes can be utilised in further studies to elucidate the regulatory mechanisms of the genes.

## Methods

### Strains, media and culture conditions

The strain used for the transcriptional profiling was *Trichoderma reesei* Rut-C30 (ATCC 56765, VTT-D-86271, [65]) obtained from VTT Culture Collection. For preparation of spore suspension, the fungus was grown on potato-dextrose plates (Difco) for 5 days. The spores were dislodged, suspended in a buffer containing 0.8% NaCl, 0.025% Tween20 and 20% glycerol, filtered through cotton, and stored at −80°C.

For the induction experiments, *T. reesei* was first cultivated on minimal medium ((NH_4_)_2_SO_4_ 7.6 g l^-1^, KH_2_PO_4_ 15.0 g l^-1^, 2.4 mM MgSO_4_^.^7H_2_O, 4.1 mM CaCl_2_^.^H_2_O, CoCl_2_ 3.7 mg l^-1^, FeSO_4_^.^7H_2_O 5 mg l^-1^, ZnSO_4_^.^7H_2_O 1.4 mg l^-1^, MnSO_4_^.^7H_2_O 1.6 mg l^-1^, pH adjusted to 4.8 with KOH) supplemented with 2% (w/v) of sorbitol as a carbon source. The medium was inoculated with 8 × 10^7^ spores per 200 ml aliquots of the medium, and cultivated in shake flasks at 28°C, with shaking at 250 rpm, until biomass dry weight in the cultures was close to 0.9 g/l (4 days). In order to get equal starting material for all the inducing conditions, the preculture aliquots were first mixed together, then divided again in 200 ml aliquots in shake flasks, and let to recover for 30 min at 28°C with shaking at 250 rpm before addition of the inducing substrates. The inducing substrates (for details, see below), suspended in 100 ml of minimal medium, were combined with the 200 ml aliquots of the preculture to start the induction, and the cultivation was continued under the same conditions (28°C, 250 rpm) for 3 days. In uninduced control cultures, 100 ml of minimal medium without inducer was added, and the control cultures were treated similarly to the induced cultures throughout the experiment. Samples for RNA isolation were collected after 0 min, 6 hours, 17 hours, 41 hours and 65 hours of the onset of the induction. The time points were selected to be long enough for induction but to minimize the possible changes in the gene expression related to the growth of the fungus. For the RNA isolation, the mycelium was collected by filtering, washed with equal volume of 0.7% NaCl, frozen immediately in liquid nitrogen, and stored at −80°C. In addition samples for determining the biomass dry weight were withdrawn from the precultures and separate uninduced control cultures during the induction. Biomass dry weight was determined by drying the mycelium samples, collected as above, to constant weight. pH of the cultures was measured throughout the cultivation.

**Figure 6:**
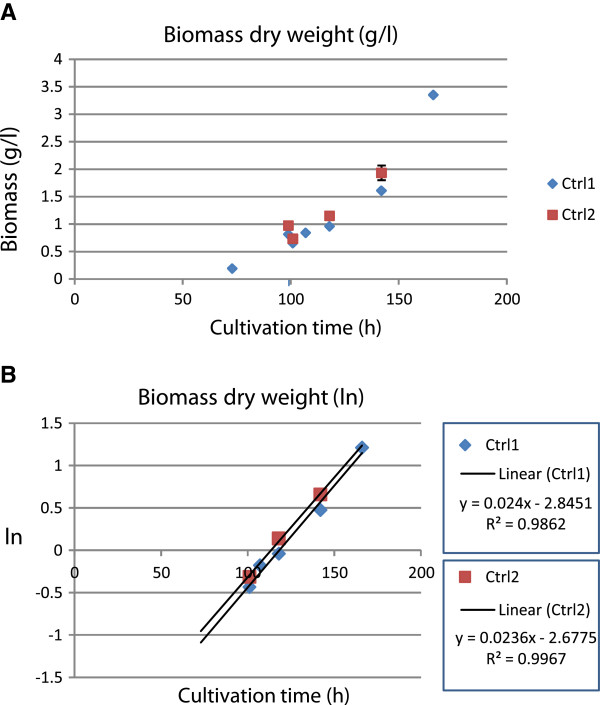
**Growth curves of the control cultures in the induction experiments.** Biomass dry weight at different time points of cultivation is shown in a linear scale (**A**), and in a logarithmic scale (**B**) for calculation of the specific growth rate. Equations for the linear trend lines of the growth curve after the induction time point, 100 h, are shown in the legend of panel B.

In this study, the induction experiment was carried out in two parts. In the first cultivation set, the inducing substrates used were 0.75% (w/v) Avicel cellulose (Fluka BioChemika), 1% (dry matter w/v) pretreated wheat straw, 1% (dry matter w/v) pretreated spruce, or 0.75 mM α-sophorose (Serva). In the second cultivation set the inducing substrates were 1% (w/v) Avicel cellulose (Fluka BioChemika), 1% (w/v) bagasse ground to homogenous composition, 1% (dry matter w/v) bagasse pretreated using steam explosion, 1% (dry matter w/v) enzymatically hydrolysed pretreated bagasse, 1% (w/v) birch xylan (Roth 7500), 1% (w/v) oat spelt xylan (Sigma-Aldrich, XO627). Uninduced control cultures were included in both cultivation sets. The inducing substrates were added at the same phase of active growth in both cultivation sets. In the first set the biomass dry weight was 0.81 g/l by the addition of the inducers, and 0.97 g/l in the second set, being less than 25% of the maximal biomass in the experiment. Biomass dry weight measurement showed that growth continued logarithmically in the control cultures of both sets during the mock induction. The same specific growth rate, 0.024/h, was measured for both cultivation sets (Figure 6). Thus, it was concluded that the induction took place at a similar growth phase in both cultivation sets. Fungal biomass dry weight could not be measured in the induced cultures due to the insoluble substrates added.

### Preparation of the inducing substrates

Steam exploded spruce was kindly provided by Guido Zacchi (Lund University, Sweden). Steam explosion had been done as in [66]. Steam exploded wheat straw was obtained from IFP Energies Nouvelles (France). Bagasse was pretreated by steam explosion using steam pressure 14.5 bar at 200°C (10 l kettle) for 5 minutes without the addition of acid or SO_2_ (kindly provided by Anne Kallioinen, VTT). The steam exploded material was washed with distilled water (1 kg/200 ml) after which the insoluble material was filtered, washed with hot tap water (1 kg/2000 ml) and filtered again to obtain an insoluble fibre fraction. The filtered spruce and bagasse materials were washed further twice with 2 l of 82°C distilled water (filtered between washes) and once with 400 ml of 85°C distilled water after which the material was filtered.

Enzymatically pretreated bagasse was obtained by incubating the washed fibre fraction of the steam exploded bagasse with the cellulase mixture Celluclast 1.5 L FG (Novozymes) (50 FPU/g cellulose in the material) and β-glucosidase Novozym 188 DCN00206 (Novozymes) (500 nkat /g cellulose in the material) in 50 mM sodium acetate buffer, pH 4.8, first for 24 h at 45°C with shaking 160 rpm. After the initial 24 h of incubation, insoluble material was collected by centrifugation (20 min, 5300 g, 20°C, Sorvall RC12BP H12000 rotor), resuspended in fresh buffer, and incubation was continued with newly added enzymes (as above) for further 48 h. After the incubation, insoluble material was recovered by centrifugation as above and washed three times with distilled water (pH adjusted to 2.5 with HCl). After the cellulase treatment, the material was resuspensed in final concentration of 5% (w/v) in 50 mM Na_2_HPO_4_, pH 6.0, and incubated with Protease N (PRW12511N, Amano) (100 nkat / substrate dry weight) for 24 h at 40°C with magnetic stirring. Insoluble fraction was collected by centrifugation, resuspended in 80°C distilled water, and incubated at 80°C for 15 min to inactivate the protease. The insoluble material was washed three times with 1 volume of distilled water.

The carbohydrate composition of the pre-treated substrates (mg/100 mg of dry matter) is shown in Table 2.

### Isolation of total RNA, preparation of cDNA, and microarray analytics

Frozen mycelium was ground under liquid nitrogen using mortar and pestle, and total RNA was isolated using Trizol reagent (Invitrogen Life Technologies, Carlsbad, CA, USA) according to manufacturer's instructions. RNA was purified using RNeasy Mini Kit (Qiagen, Hilden, Germany) and RNA concentration was measured using NanoDrop ND-1000 (NanoDrop Technologies Inc. Wilmington, DE, USA). Integrity of RNA was analysed using Agilent 2100 Bioanalyzer (Agilent Technologies, Palo Alto, CA, USA).

Microarray analysis of total RNA isolated from the first cultivation set (with Avicel, wheat straw, spruce and sophorose as inducing substrates) was carried out by Roche NimbleGen (Roche‐NimbleGen, Inc., Madison, WI, USA) as part of their array service, including the synthesis and labelling of cDNA, hybridization of cDNA on microarray slides, and scanning of the slides to produce the raw data files. For the microarray analysis of the second cultivation set, the total RNA samples were processed essentially according to the instructions by RocheNimblegen. The double-stranded cDNA was synthesised using Superscript Double-Stranded cDNA synthesis Kit (Invitrogen), and the integrity of the double-stranded cDNA was analyzed using Agilent 2100 Bioanalyzer (Agilent Technologies, Palo Alto, CA, USA). The double-stranded cDNA was labelled with Cy3 fluorescent dye, hybridized to microarray slides (Roche‐NimbleGen, Inc., Madison, WI, USA) and scanned using Roche NimbleGen Microarray scanner according to the instructions of the manufacturer.

The probe design and manufacturing of the microarray slides was carried out by RocheNimbleGen. For the first cultivation set the design was based on the *T. reesei* genome version 1.2 [47] as described in [67]. For the second cultivation set, an array design based on the *T. reesei* genome version 2.0 [38] was used. In the latter array format, six 60mer probes were designed for each of the genes.

The microarray data was analysed using the R package Oligo for preprocessing of the data and the package Limma for identifying differentially expressed genes [68-70]. In the analysis of the differentially expressed genes, the signals in the samples of the induced cultures were compared to the ones of uninduced control cultures at the corresponding time point. Four biological replicates were analysed for each condition and each time point. The cut-off used for statistical significance was p-value < 0.01, and an additional cut-off for the log2-scale fold change (>0.4) was set.

### Quantitative PCR

Total RNA isolated from samples collected at the induction time point 17 h were subjected to qPCR analysis of a selected set of genes. cDNA was synthesized using Transcriptor High Fidelity cDNA synthesis kit (Roche), 2 μg of total RNA as a template. A dilution of 1:100 from the cDNA sample was used for assays. qPCR reaction was performed using LightCycler 480 SYBR Green I Master kit (Roche) and Light Cycler 480 II instrument according to the instructions of the manufacturer. The primers used in the qPCR are listed in Table 3. The results were analysed with LightCycler 480 Software release 1.5.0. (version 1.5.0.39) using *gpd1* signal for normalisation. The results are shown as a fold change as compared to the uninduced control cultures.

**Table 3:** Primers used in the quantitative PCR method

**Gene**	**5' forward primer**	**3' reverse primer**
*cbh1*	GCGGATCCTCTTTCTCAG	ATGTTGGCGTAGTAATCATCC
*cbh2*	TCCTGGTTATTGAGCCTGAC	GCAACATTTGGAAGGTTCAG
*egl1*	GTCTACTACGAACTCGAC	GTAGTAGTCGTTGCTATACTG
*egl2*	CTGTACCACAGATGGCAC	ATCATACTTGGAAATGCTCG
*xyn1*	AAACTACCAAACTGGCGG	TTGATGGGAGCAGAAGATCC
*xyn2*	CGGCTACTTCTACTCGTACTG	TTGATGACCTTGTTCTTGGTG
*xyn4*	TTTGACATTGCGACATGGC	GCCGCTATAATCCCAGGT
*abf1*	ATATCCTTCCGATGCAACAG	AGAGATTGACGAACCGAC
*axe1*	TAAAGCAGCAATCTTCATGG	GCAGTAAGACTTGATCTTGG
*cel3c*	ACATCAAGCATTTCATCGCC	ACACTATCCATAAAGGGCCA
*cel3d*	AGCATATCTCAACTACGCCA	GAAGGTAGCGTAAGACAGG
*bxl1*	GTCACTCTTCCAAGCTCAG	ATCGTTACCTCTTCTCCCA
*cel61b*	TGAACTTCTTGCTGCCCA	TAGAGCTGAGTTGCAGGAG
*xyn3*	TACAAGGGCAAGATTCGTG	ACTGGCTTCCAATACCGT
*gpd1*	TCCATTCGTGTCCCTACC	AGATACCAGCCTCAATGTC
*swo1*	ATTACTACACCCAATTCTGGTC	GACAGCCGTATTTGAAGTC

### Mapping *T. reesei* proteome to CAZy database

Information in the CAZy database [5,6] was downloaded family by family (November 2010). The sequence information of reported CAZy family members was then downloaded from NCBI [71] using the database identifier listed on CAZy site. For sequences that did not have a NCBI idenfier, a Uniprot identifier was used instead. Sequences that did not have either identifier were left out. Finally, a searchable BLAST database was created from the protein sequence information using formatdb command from the BLAST program suite [48]. The information on the CAZy family annotation was retained during the construction of the local CAZy BLAST database. The local CAZy BLAST database was queried with each *T. reesei* protein using blastp [48]. Only blast matches having E-value smaller than 10^-11^ were retained.

Each *T. reesei* gene with significant similarity to CAZy database genes was mapped to protein homology clusters described in [49] and updated to include 49 fungi [50], and all the protein members of the found clusters were mapped to CAZy by blastp [48]. A cluster member protein was identified as a CAZyme (carbohydrate active enzyme) if it had a hit in CAZy dabase of at least 97% identity covering over 200 amino acids in the blastp alignment (Additional file 2).

Interpro protein domains [55] from all protein sequences were predicted using InterproScan [72].

### Phylogenetic analysis of *T. reesei* CAZymes

Reconstruction of phylograms of protein homology clusters was carried out as in [73] i.e. the sequences of the proteins in the clusters were aligned with MAFFT [74,75], the alignments trimmed with trimAL [76] and phylogenetic trees constructed with RAxML version 7.2.8 [77], except that due to the large number of trees only 100 bootstraps per tree were made. Trees were visualised using the R [78] library ape [79].

### Aligning *T. reesei* CAZymes against the PFAM profiles of a CAZy family

Sequences belonging to selected CAZy families were clustered to help annotate *T. reesei* proteins belonging to the family. The members of the CAZy family, including *T. reesei* candidates, were aligned to the PFAM [80] profile of the family using hmmalign from the HMMer program package [81]. The alignment was then fed into the same pipeline that was used for the construction of phylograms of protein homology clusters. Namely, the alignment was trimmed with trimAL [76] and phylogenetic trees constructed with RAxML version 7.2.8 [77], except that due to the large number of trees only 100 bootstraps per tree were made. Typically the sequences grouped in the phylogenetic tree by order (fungal, bacterial) and by function. Annotation of *T. reesei* proteins was conducted by studying the members of the CAZy family assigned to the same branch as the *T. reesei* protein and by studying whether the *T. reesei* protein contained the conserved amino acids typical for the members of the CAZy family.

## Abbreviations

CAZy: Carbohydrate active enzyme database; CAZyme: Carbohydrate active enzyme; CBM: Cellulose binding module; CE: Carbohydrate esterase; GH: Glycoside hydrolase; GT: Glycosyl transferase; PL: Polysaccharide lyase.

## Competing interest

The authors declare that they have no competing interests.

## Authors' contributions

MH carried out fungal cultivations and microarray detection of the expression signals, and participitated in the phylogenetic analysis of CAZyme genes, annotation of the CAZymes as well as in the analysis and interpretation of the microarray data, and drafted the manuscript, MA and MO participated in designing the computational analysis required for gene annotations and carried out mapping of *T. reesei* proteome to CAZy database and phylogenetic analysis of the CAZyme genes, NA carried out qPCR analysis of transcript levels, MP and MS conceived of the study, participated in its design and coordination, and TMP participated in the design and coordination of the study, carried out microarray data analysis, and helped to draft the manuscript. All authors read and approved the final manuscript.

## Supplementary Material

Additional file 1 **Results of mapping the *T. reesei *proteome to the CAZy database.** Results shown are the best blast matches for the genes. Results have been sorted according to the e value. Columns C-L are from the default output of blastp search. Column M has the gene descriptions from the CAZy database with the possible EC numbers and column B has the same information as column M with also the possible CAZy family and information whether the protein is characterized (cha) and if its structure has been determined (str). Click here for file

Additional file 2 **Cut-offs of mapping protein sequences to CAZy database member proteins.** (A) Scatterplot of blastp results of all protein sequences from protein clusters of 49 fungi with a *T. reesei* candidate CAZyme. Only values for best hit are shown. Each sequence is represented by the majority vote predicted CAZy family identifier of the protein cluster. Y axis shows the identity percentage from blastp alignment and X axis the length of the alignment as amino acids. Protein was said to be found in CAZy if it had a hit of at least 97% identity which covered over 200 amino acids. (B) Scatterplot of blastp results of protein cluster averages of protein clusters with a CAZy database protein. For each protein only the value of the best hit was considered for counting the cluster averages. Each cluster is represented by the majority vote predicted CAZy family identifier of the protein cluster. Y axis shows the average identity percentage from blastp alignment and X axis the length of the alignment as amino acids. Clusters above the red line and shown in red were accepted for further analysis. (C) Scatterplot of protein cluster averages of protein clusters without a CAZy database protein. See further details from panel B. Click here for file

Additional file 3 **Annotation of *T. reesei *CAZymes.***T. reesei* glycoside hydrolase, carbohydrate esterase (excluding CE10) and polysaccharide lyase genes, the annotation of the genes and the bases used for annotation. (a), gene identifier as in *T. reesei* v2.0 data base [38]; (b), name given to the gene in the publication/data base marked in the reference column; (c), reference to previous studies or to *T. reesei* database versions 1.2 and 2.0. (“A”, a previous annotation has been specified/updated during this study); (d), other names used for the gene in marked references; (e), annotation given for the gene in *T. reesei* v2.0 data base; (f), best match for the *T. reesei* CAZyme when *T. reesei* proteome was mapped with blast search to the protein sequences of the CAZy database. The gi identifiers refer to the NCBI protein database; (g), best characterized match for the *T. reesei* CAZyme when *T. reesei* proteome was mapped with blast search to the protein sequences of the CAZy database. The gi identifiers refer to the NCBI protein database.; (h), protein cluster the *T. reesei* CAZyme was assigned to when the protein clusters were mapped to CAZy database by a blast search; (i), functional subgroups within the protein cluster determined according to phylogenetic analysis; (j), characterized protein from another fungus and/or other *T. reesei* proteins closest to the *T. reesei* CAZyme in a phylogenetic tree constructed from the members of a protein cluster. Uniprot protein identifier is preceded by a code that specifies the species (Additional file 5); (k), CAZy family assigned to the *T. reesei* CAZyme based on the CAZy family members present in the protein cluster; (l), existence of a functional domain/domains for the *T. reesei* CAZyme that supports the CAZy prediction. All the functional domains of the *T. reesei* CAZymes are found from Additional file 4; (m), protein closest to the *T. reesei* CAZyme in a phylogenetic tree constructed from alignment against PFAM of a CAZy family. Protein identifier is preceded by a six letter code that specifies the species and a possible EC number of the enzyme is given. Fusequ = *Fusarium equiseti*, Coccar = *Cochliobolus carbonum*, Hyplix = *Hypocrea lixii*, Bifbif = *Bifidobacterium bifidum*, Acrimp = *Acremonium implicatum*, Penchr = *Penicillium chrysogenum*, Phachr = *Phanerochaete chrysosporium*, Maggri = *Magnaporthe grisea*, Glolin = *Glomerella lindemuthiana*, Aspnid = *Aspergillus nidulans*, Hypvir = *Hypocrea virens*, Flacol = *Flavobacterium columnare*, Aspfum = *Aspergillus fumigatus*, Azocau = *Azorhizobium caulinodans*, Enthis = *Entamoeba histolytica*, Hypjec = *Hypocrea jecorina*, Clothe = *Clostridium thermocellum.* Click here for file

Additional file 4 **Functional Interpro domains of *T. reesei *CAZymes.** Gene identifiers are as in *T. reesei* database 2.0 [38]. Domain identifiers and annotations are as in InterPro database [55]. Click here for file

Additional file 5 **Fungal species from the protein clusters.** Abbreviations specifying the 49 fungal species belonging to the protein homology clusters and the taxonomy of the species. Click here for file

Additional file 6 **Phylogenetic trees for *T. reesei *CAZymes.** Trees are constructed from the protein clusters of 49 fungi including *T. reesei* CAZymes. Proteins are named with an uniprot protein identifier which is preceded by a code that specifies the species (Additional file 5). Click here for file

Additional file 7 **Heatmap comparing the protein cluster content of different fungi.** Each row is a protein cluster (marked with T and a number) and each column is a fungal species. The colouring of the cells is proportional to the count of proteins. A phylogram of the species is shown above the heatmap together with a colour bar coloured by the taxon of the species. Species abbreviations below the heatmap are explained in Additional file 5. On the left, the colour bar named IDp shows the identity percentage of the genes belonging to the same protein cluster. The darker the colour, the more identical the proteins are. Click here for file

Additional file 8 **Phylogeny of *T. reesei *CAZyme gene 59791.** Tree was constructed from the results of blastp against the non-redundant proteinsequences database [82] using BLAST pairwise alignment. The tree method used was fast minimum evolution. Maximum sequence difference was 0.85 and distance model used was Grishin. Click here for file

Additional file 9 **Phylogeny of *T. reesei *CAZyme gene 73101.** Tree was constructed from the results of blastp against the non-redundant proteinsequences database [82] using BLAST pairwise alignment. The tree method used was fast minimum evolution. Maximum sequence difference was 0.85 and distance model used was Grishin. Click here for file

Additional file 10 **Phylogeny of *T. reesei *CAZyme gene 108671.** Tree was constructed from the results of blastp against the non-redundant proteinsequences database [82] using BLAST pairwise alignment. The tree method used was fast minimum evolution. Maximum sequence difference was 0.85 and distance model used was Grishin. Click here for file

Additional file 11 **Fold changes, signal intensities and significance test for the differential expression of *T. reesei *CAZyme genes.** BO, ground bagasse; BS, steam exploded bagasse; BE, enzymatically hydrolysed steam exploded bagasse; XO, oat spelt xylan; XB, birch xylan; AV1, 1% Avicel cellulose; AV0.75, 0.75% Avicel cellulose; WH, steam exploded wheat straw; SP, steam exploded spruce; SO, sophorose; CO1: uninduced control from the first cultivation set; CO2, uninduced control from the second cultivation. Columns marked “Fold change” show the fold change of the signal in the induced culture vs. the signal in the uninduced cultures at the time point (log2 scale). The intensity of the red colour and blue colour indicates the strength of positive and negative fold changes, respectively. Columns marked “Significance” show the results of a significance test (R package limma, p-value < 0.01, log2 fold change > 0.4), 1 indicates induction and −1 repression. Columns marked “Signal intensity” show the signal intensities (log2 scale) from the microarray analysis. Colours indicate different intensities of signals, red represents the strongest signals and green the weakest signals. (a), gene identifier as in *T. reesei* v2.0 data base [38]; (b), class of the protein according to the CAZy classification [5]; (c), family of the protein according to the CAZy classification; (d), protein cluster the *T. reesei* CAZyme was assigned to when the protein clusters were mapped to CAZy database by a blast search; (e), functional subgroups within the protein cluster determined according to phylogenetic analysis; (f), heat map branch (A-R) the gene was assigned to according to the expression profile of the gene; (g), the order in which the genes appear in the heat map representing the expression profiles; (h), induction of the gene by the presence of any of the substrates tested in the study is indicated by “1”. Click here for file

Additional file 12 **Comparison of relative transcript signals obtained using microarray or qPCR detection.** Pre-processed and normalised microarray signals (log2 scale) were plotted against the relative expression signals obtained using qPCR analysis of the same samples (shown as -ΔCp, normalised using the signals of *gpd1*). Expression data of the genes *xyn4, xyn3, egl2, cel61b, bxl1, egl1, abf1, xyn2, cbh1, swo1, cel3d, cbh2, axe1, xyn1* and *cel3c* were combined and plotted. Click here for file
