# Supplementary material for: Re-annotation of the CAZy genes of Trichoderma reesei and transcription in the presence of lignocellulosic substrates
Source: Microb Cell Fact. 2012 Oct 4;11:134. doi: 10.1186/1475-2859-11-134 (PMC3526510; doi:10.1186/1475-2859-11-134)
Supplement: Additional file 2 — Cut-offs of mapping protein sequences to CAZy database member proteins. (A) Scatterplot of blastp results of all protein sequences from protein clusters of 49 fungi with a T. reesei candidate CAZyme. Only values for best hit are shown. Each sequence is represented by the majority vote predicted CAZy family identifier of the protein cluster. Y axis shows the identity percentage from blastp alignment and X axis the length of the alignment as amino acids. Protein was said to be found in CAZy if it had a hit of at least 97% identity which covered over 200 amino acids. (B) Scatterplot of blastp results of protein cluster averages of protein clusters with a CAZy database protein. For each protein only the value of the best hit was considered for counting the cluster averages. Each cluster is represented by the majority vote predicted CAZy family identifier of the protein cluster. Y axis shows the average identity percentage from blastp alignment and X axis the length of the alignment as amino acids. Clusters above the red line and shown in red were accepted for further analysis. (C) Scatterplot of protein cluster averages of protein clusters without a CAZy database protein. See further details from panel B. [file 1475-2859-11-134-S2.pdf]

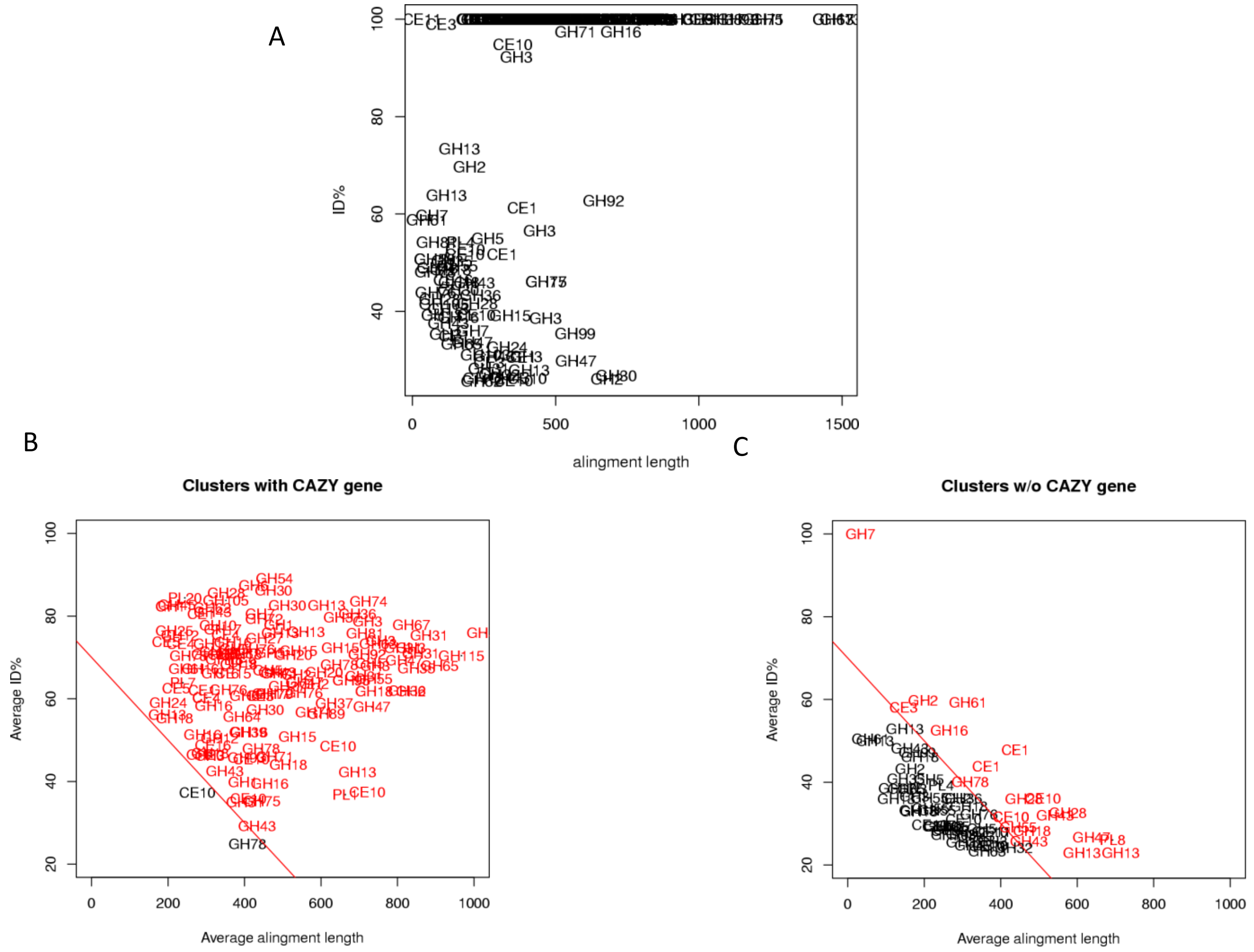

Additional file 2. Cut-offs of mapping protein sequences to CAZY database member proteins. (A) Scatterplot of blastp results of all protein sequences from protein clusters of 49 fungi with a *T. reesei* candidate CAZyme gene. Only values for best hit are shown. Each sequence is represented by the majority vote predicted CAZY family identifier of the protein cluster. Y axis shows the identity percentage from blastp alignment and X axis the length of the alignment as amino acids. Protein was said to be found in CAZY if it had a hit of at least 97% identity which covered over 200 amino acids. (B) Scatterplot of blastp results of protein cluster averages of protein clusters with a CAZY database protein. For each protein only the value of the best hit was considered for counting the cluster averages. Each cluster is represented by the majority vote predicted CAZY family identifier of the protein cluster. Y axis shows the average identity percentage from blastp alignment and X axis the length of the alignment as amino acids. Clusters above the red line and shown in red were accepted for further analysis. (C) Scatterplot of protein cluster averages of protein clusters without a CAZY database protein. See further details from panel B.
