# Supplementary material for: Re-annotation of the CAZy genes of Trichoderma reesei and transcription in the presence of lignocellulosic substrates
Source: Microb Cell Fact. 2012 Oct 4;11:134. doi: 10.1186/1475-2859-11-134 (PMC3526510; doi:10.1186/1475-2859-11-134)
Supplement: Additional file 6 — Phylogenetic trees for T. reesei CAZymes. Trees are constructed from the protein clusters of 49 fungi including T. reesei CAZymes. Proteins are named with an uniprot protein identifier which is preceded by a code that specifies the species (Additional file 5). [file 1475-2859-11-134-S6.zip › Additional file 5/index.html]

  

## Supplementary phylograms for H�kkinen & al 2012

- Glycoside hydrolase 7: Protein cluster 470
- Glycoside hydrolase 5: Protein cluster 778
- Glycoside hydrolase 61: Protein cluster 77
- Glycoside hydrolase 61: Protein cluster 515
- Glycoside hydrolase 1: Protein cluster 662
- Glycoside hydrolase 3: Protein cluster 110
- Glycoside hydrolase 3: Protein cluster 132
- Glycoside hydrolase 3: Protein cluster 3274
- Glycoside hydrolase 54: Protein cluster 5126
- Glycoside hydrolase 11: Protein cluster 643
- Glycoside hydrolase 30: Protein cluster 6176
- Carbohydrate esterase 5: Protein cluster 865
- Glycoside hydrolase 2: Protein cluster 609
- Glycoside hydrolase 27: Protein cluster 617
- Glycoside hydrolase 27: Protein cluster 12458
- Carbohydrate esterase 16: Protein cluster 3851
- Polysaccharide lyase 20: Protein cluster 5536
- Glycoside hydrolase 18: Protein cluster 115
- Glycoside hydrolase 18: Protein cluster 410
- Glycoside hydrolase 18: Protein cluster 3553
- Glycoside hydrolase 18: Protein cluster 200

### Glycoside hydrolase 7: Protein cluster 470

  

### Glycoside hydrolase 5: Protein cluster 778

  

### Glycoside hydrolase 61: Protein cluster 77

  

### Glycoside hydrolase 61: Protein cluster 515

  

### Glycoside hydrolase 1: Protein cluster 662

  

### Glycoside hydrolase 3: Protein cluster 110

  

### Glycoside hydrolase 3: Protein cluster 132

  

### Glycoside hydrolase 3: Protein cluster 3274

  

### Glycoside hydrolase 54: Protein cluster 5126

  

### Glycoside hydrolase 11: Protein cluster 643

  

### Glycoside hydrolase 30: Protein cluster 6176

  

### Carbohydrate esterase 5: Protein cluster 865

  

### Glycoside hydrolase 2: Protein cluster 609

  

### Glycoside hydrolase 27: Protein cluster 617

  

### Glycoside hydrolase 27: Protein cluster 12458

  

### Carbohydrate esterase 16: Protein cluster 3851

  

### Polysaccharide lyase 20: Protein cluster 5536

  

### Glycoside hydrolase 18: Protein cluster 115

  

### Glycoside hydrolase 18: Protein cluster 410

  

### Glycoside hydrolase 18: Protein cluster 3553

  

### Glycoside hydrolase 18: Protein cluster 200

  
