# Supplementary figures and images for: Re-annotation of the CAZy genes of Trichoderma reesei and transcription in the presence of lignocellulosic substrates
Source: Microb Cell Fact. 2012 Oct 4;11:134. doi: 10.1186/1475-2859-11-134 (PMC3526510; doi:10.1186/1475-2859-11-134)

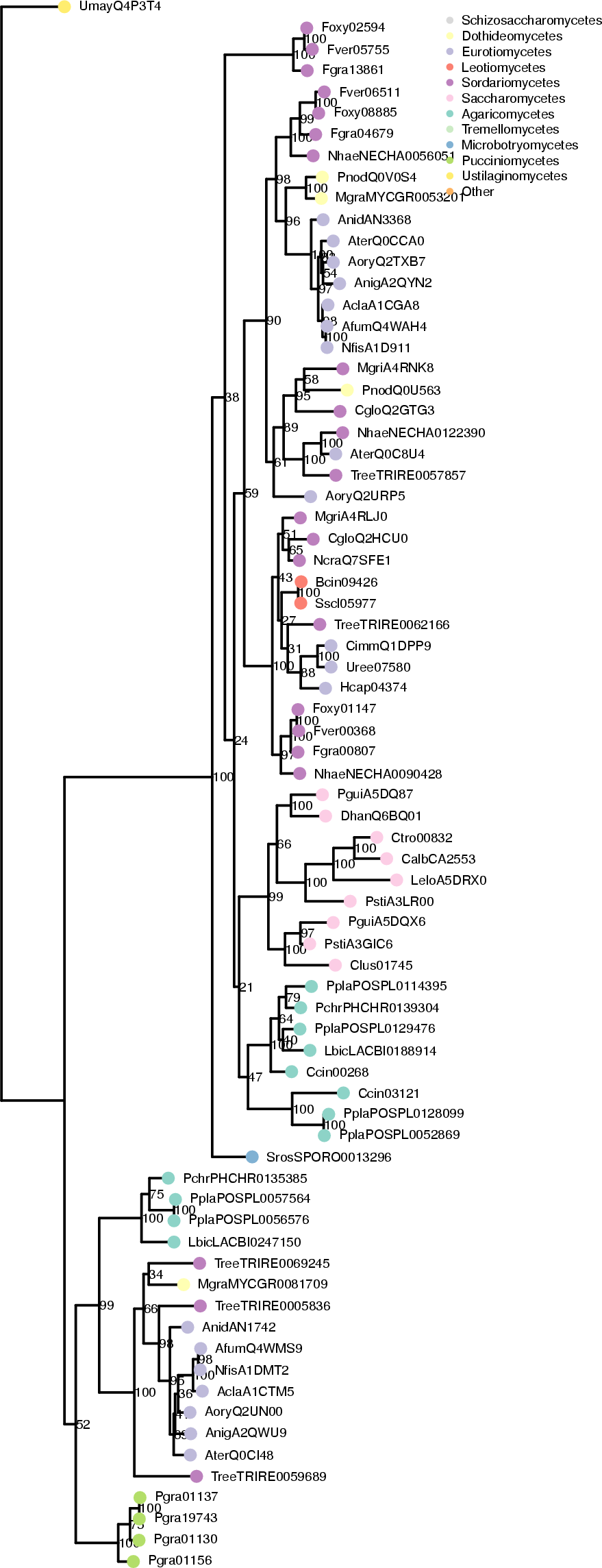

Supplement: Additional file 6 — Phylogenetic trees for T. reesei CAZymes. Trees are constructed from the protein clusters of 49 fungi including T. reesei CAZymes. Proteins are named with an uniprot protein identifier which is preceded by a code that specifies the species (Additional file 5). [file 1475-2859-11-134-S6.zip › Additional file 5/TRIRE0005836.eps.png]

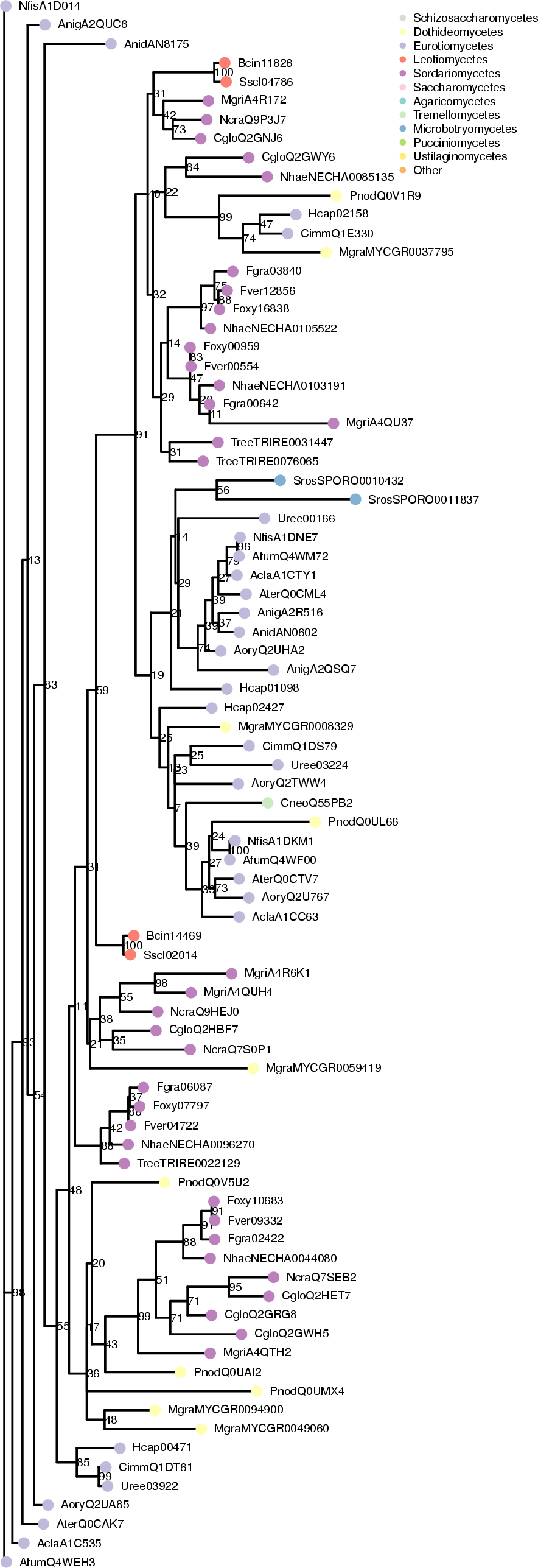

Supplement: Additional file 6 — Phylogenetic trees for T. reesei CAZymes. Trees are constructed from the protein clusters of 49 fungi including T. reesei CAZymes. Proteins are named with an uniprot protein identifier which is preceded by a code that specifies the species (Additional file 5). [file 1475-2859-11-134-S6.zip › Additional file 5/TRIRE0022129.eps.png]

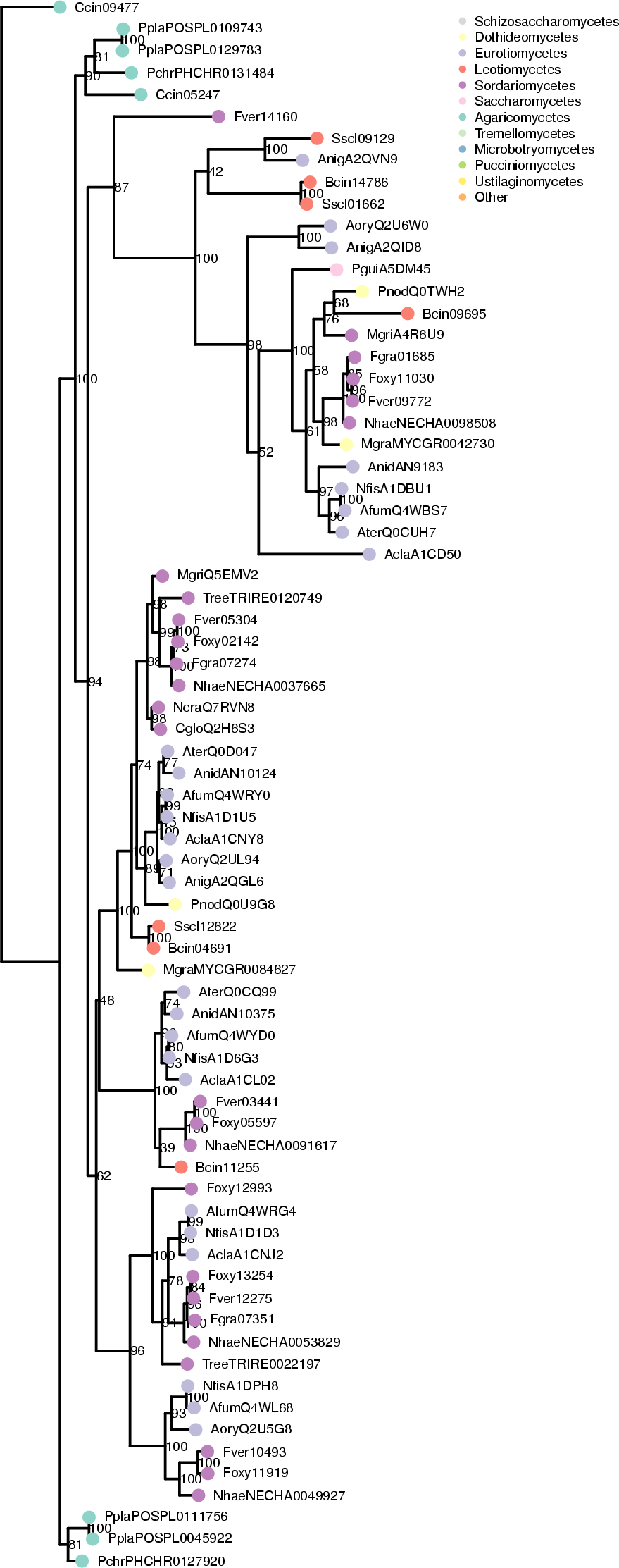

Supplement: Additional file 6 — Phylogenetic trees for T. reesei CAZymes. Trees are constructed from the protein clusters of 49 fungi including T. reesei CAZymes. Proteins are named with an uniprot protein identifier which is preceded by a code that specifies the species (Additional file 5). [file 1475-2859-11-134-S6.zip › Additional file 5/TRIRE0022197.eps.png]

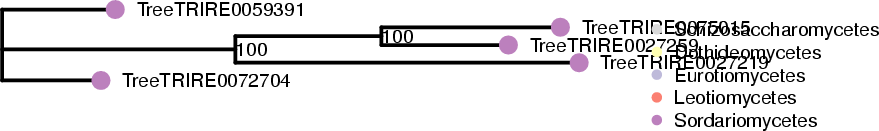

Supplement: Additional file 6 — Phylogenetic trees for T. reesei CAZymes. Trees are constructed from the protein clusters of 49 fungi including T. reesei CAZymes. Proteins are named with an uniprot protein identifier which is preceded by a code that specifies the species (Additional file 5). [file 1475-2859-11-134-S6.zip › Additional file 5/TRIRE0027219.eps.png]

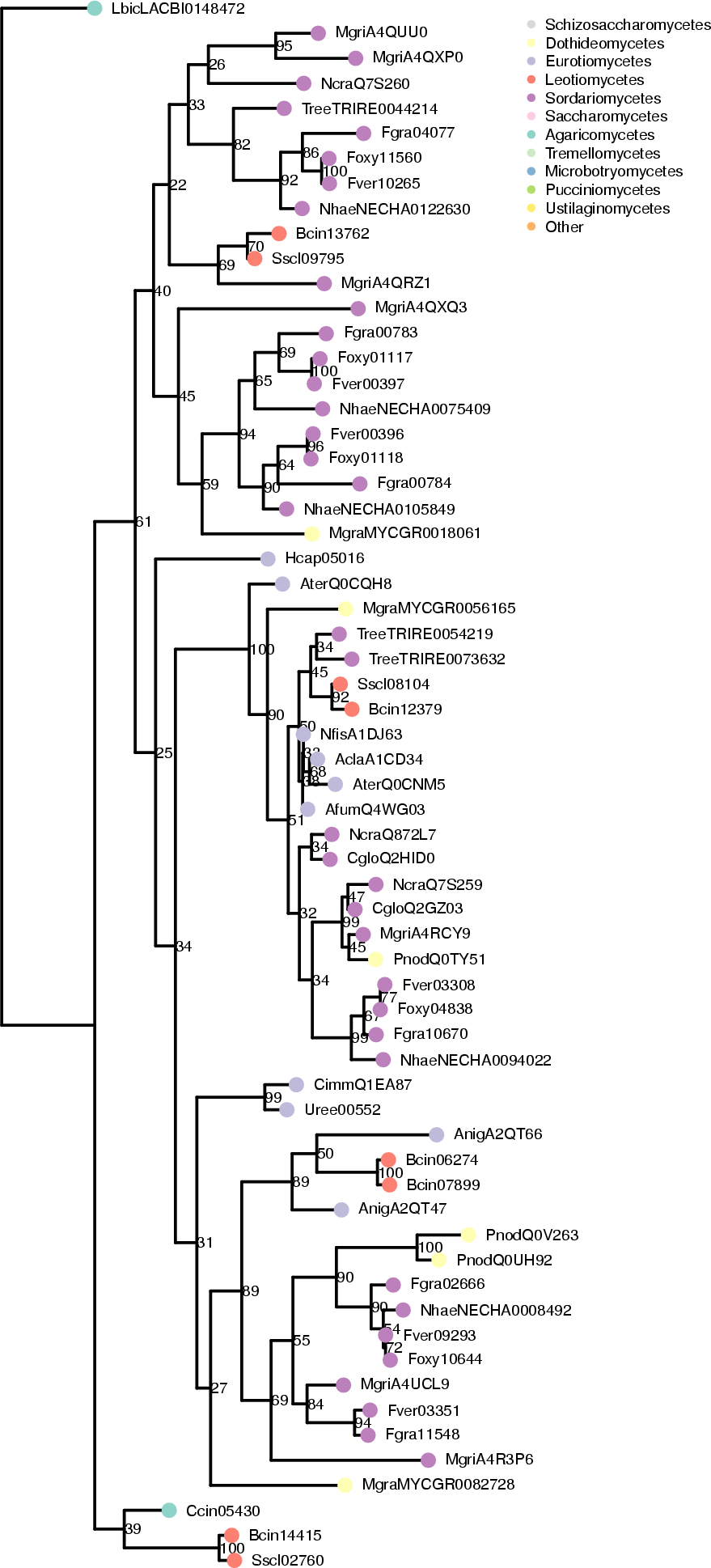

Supplement: Additional file 6 — Phylogenetic trees for T. reesei CAZymes. Trees are constructed from the protein clusters of 49 fungi including T. reesei CAZymes. Proteins are named with an uniprot protein identifier which is preceded by a code that specifies the species (Additional file 5). [file 1475-2859-11-134-S6.zip › Additional file 5/TRIRE0054219.eps.png]

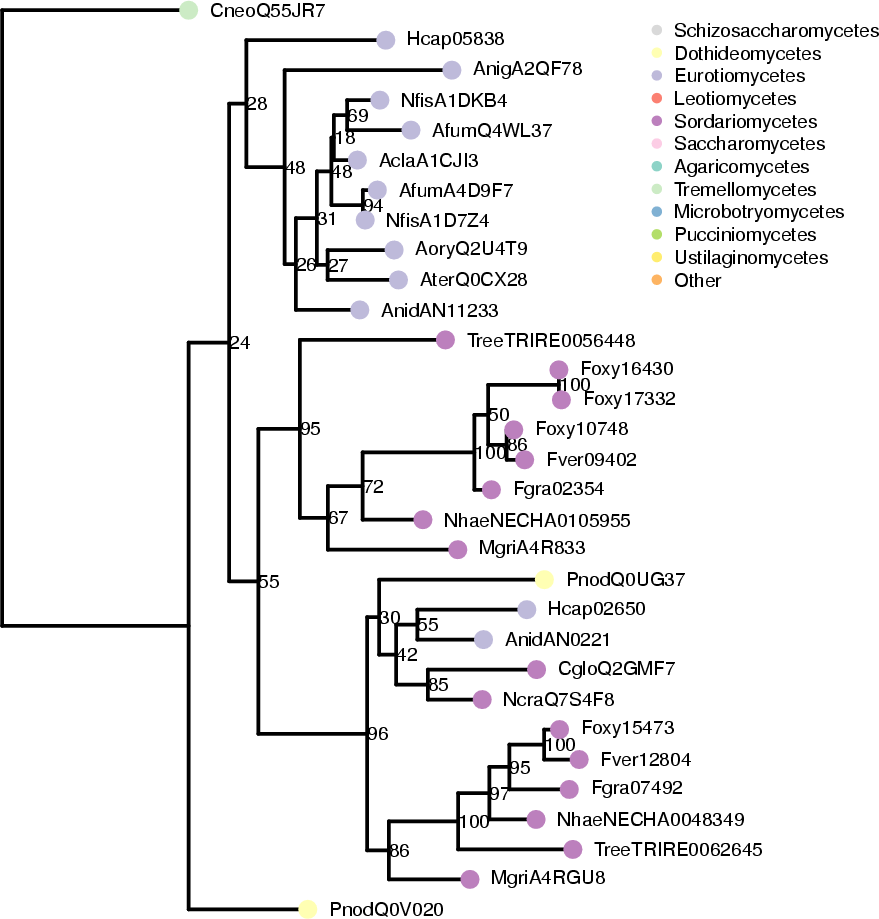

Supplement: Additional file 6 — Phylogenetic trees for T. reesei CAZymes. Trees are constructed from the protein clusters of 49 fungi including T. reesei CAZymes. Proteins are named with an uniprot protein identifier which is preceded by a code that specifies the species (Additional file 5). [file 1475-2859-11-134-S6.zip › Additional file 5/TRIRE0056448.eps.png]

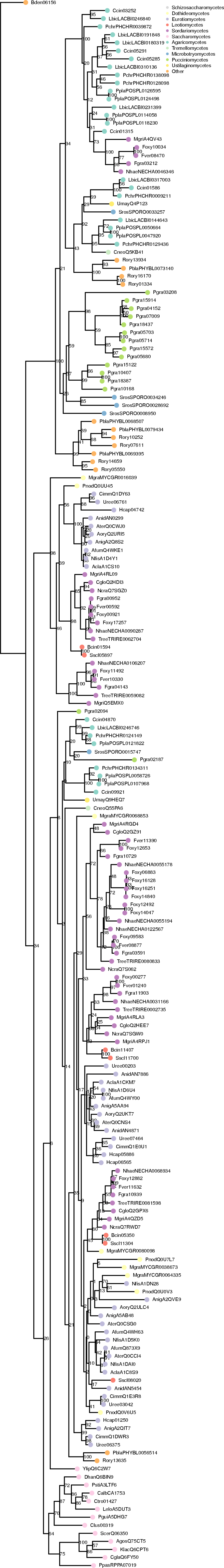

Supplement: Additional file 6 — Phylogenetic trees for T. reesei CAZymes. Trees are constructed from the protein clusters of 49 fungi including T. reesei CAZymes. Proteins are named with an uniprot protein identifier which is preceded by a code that specifies the species (Additional file 5). [file 1475-2859-11-134-S6.zip › Additional file 5/TRIRE0059082.eps.png]

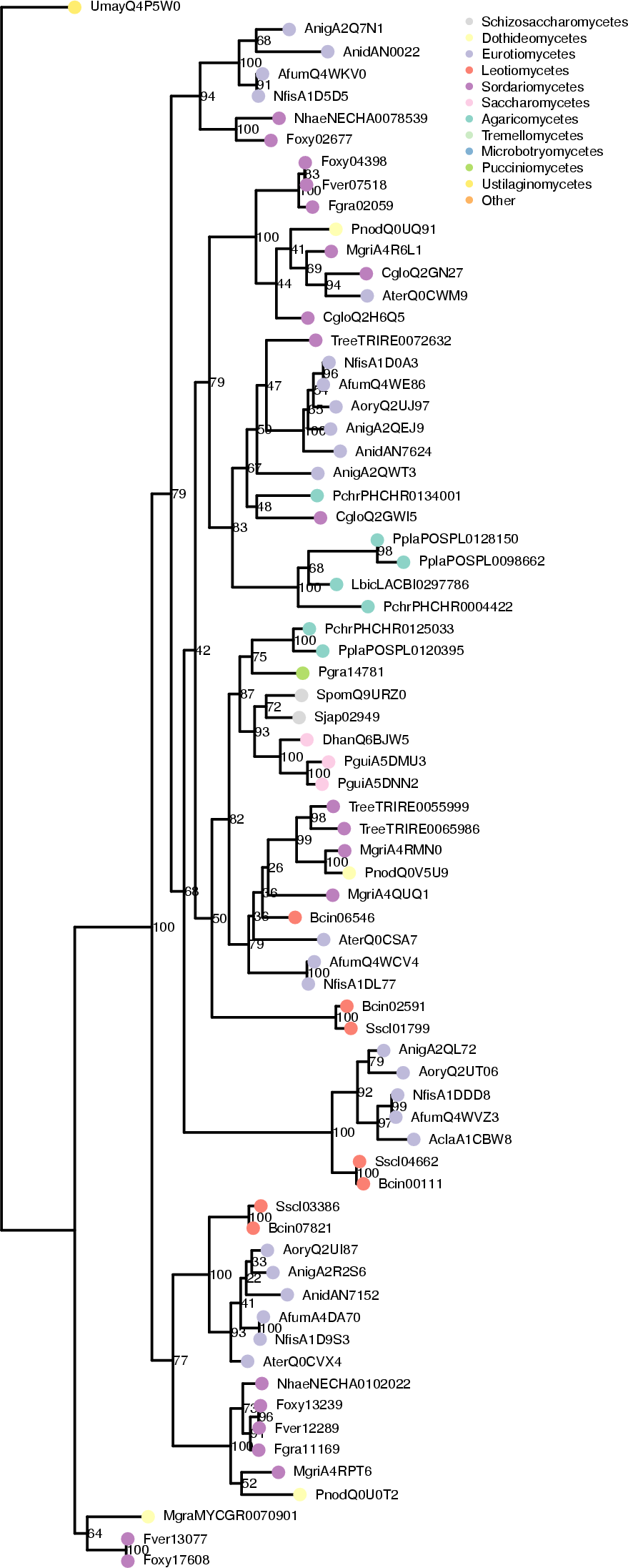

Supplement: Additional file 6 — Phylogenetic trees for T. reesei CAZymes. Trees are constructed from the protein clusters of 49 fungi including T. reesei CAZymes. Proteins are named with an uniprot protein identifier which is preceded by a code that specifies the species (Additional file 5). [file 1475-2859-11-134-S6.zip › Additional file 5/TRIRE0065986.eps.png]

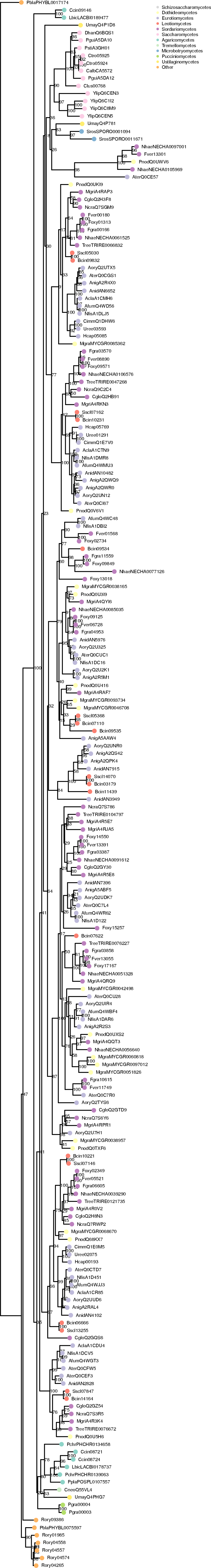

Supplement: Additional file 6 — Phylogenetic trees for T. reesei CAZymes. Trees are constructed from the protein clusters of 49 fungi including T. reesei CAZymes. Proteins are named with an uniprot protein identifier which is preceded by a code that specifies the species (Additional file 5). [file 1475-2859-11-134-S6.zip › Additional file 5/TRIRE0066832.eps.png]

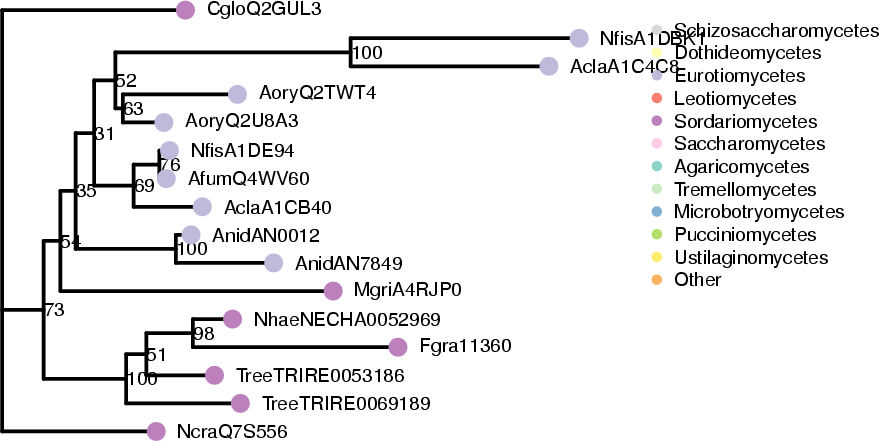

Supplement: Additional file 6 — Phylogenetic trees for T. reesei CAZymes. Trees are constructed from the protein clusters of 49 fungi including T. reesei CAZymes. Proteins are named with an uniprot protein identifier which is preceded by a code that specifies the species (Additional file 5). [file 1475-2859-11-134-S6.zip › Additional file 5/TRIRE0069189.eps.png]

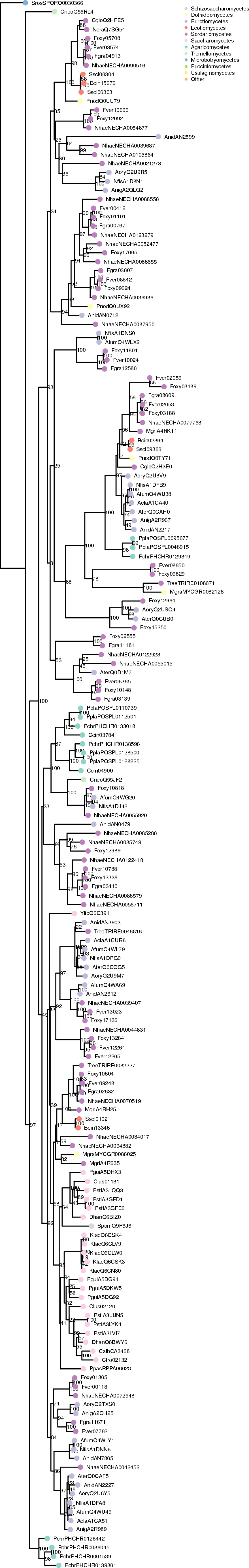

Supplement: Additional file 6 — Phylogenetic trees for T. reesei CAZymes. Trees are constructed from the protein clusters of 49 fungi including T. reesei CAZymes. Proteins are named with an uniprot protein identifier which is preceded by a code that specifies the species (Additional file 5). [file 1475-2859-11-134-S6.zip › Additional file 5/TRIRE0082227.eps.png]

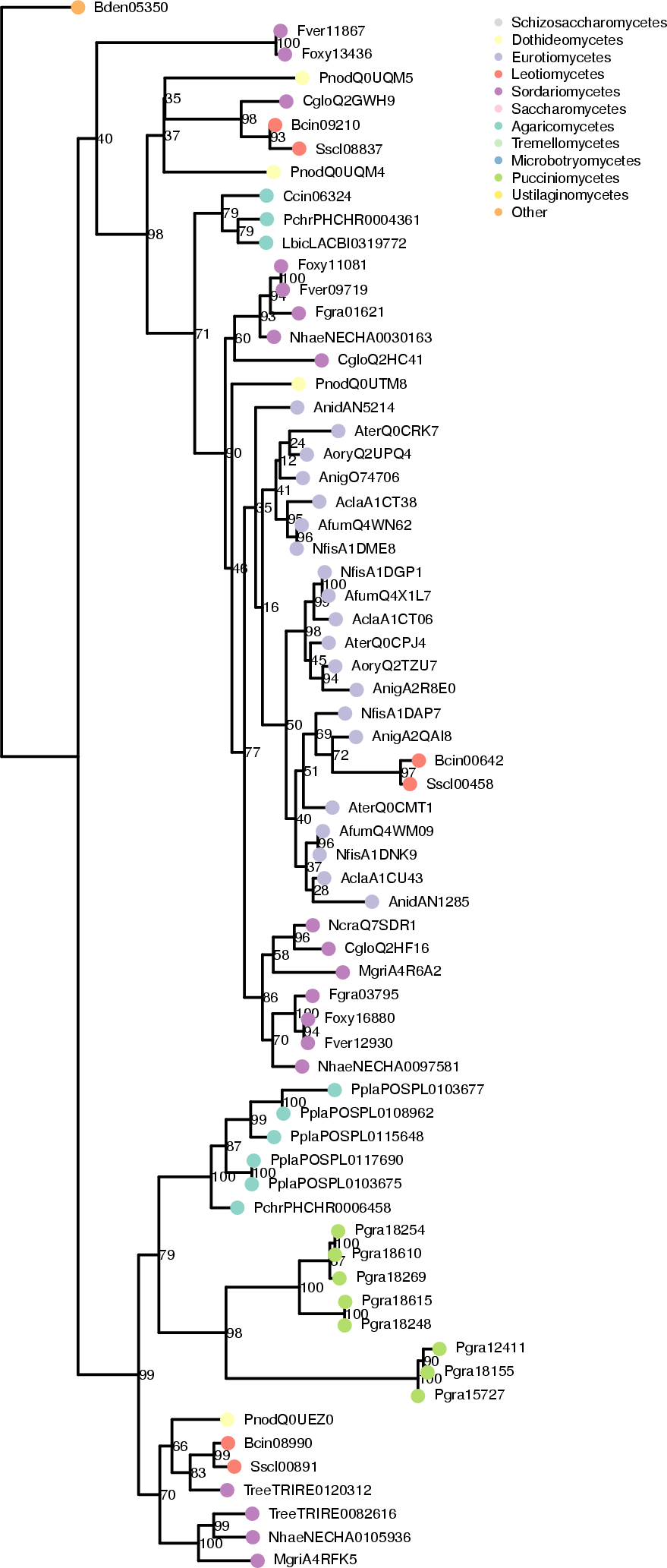

Supplement: Additional file 6 — Phylogenetic trees for T. reesei CAZymes. Trees are constructed from the protein clusters of 49 fungi including T. reesei CAZymes. Proteins are named with an uniprot protein identifier which is preceded by a code that specifies the species (Additional file 5). [file 1475-2859-11-134-S6.zip › Additional file 5/TRIRE0082616.eps.png]

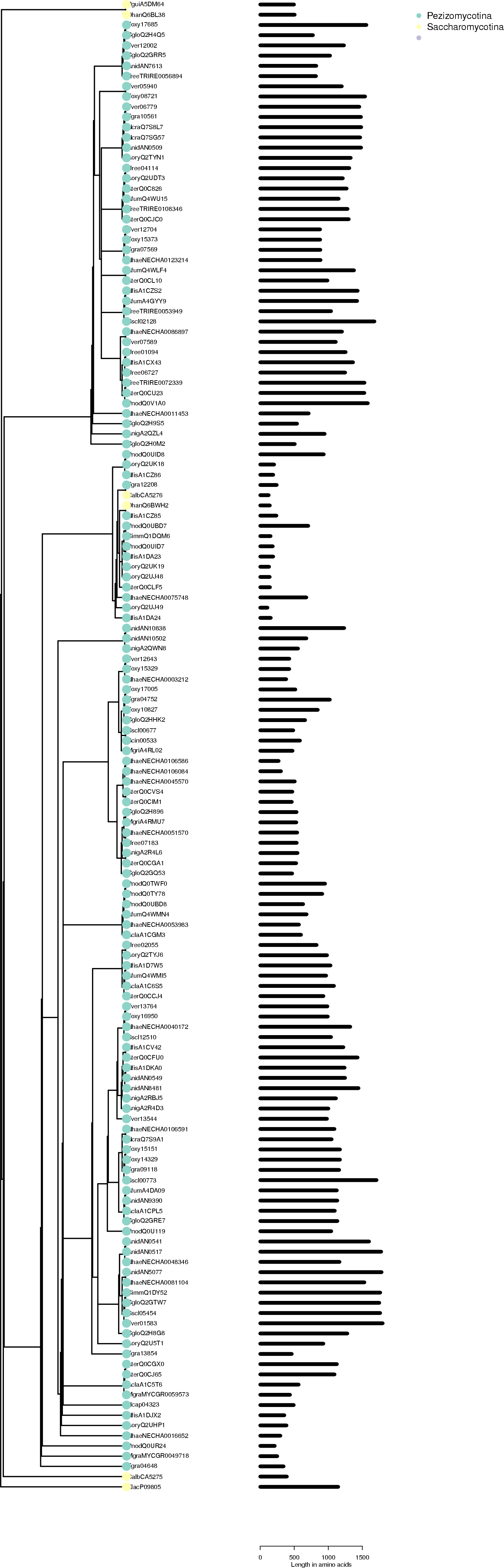

Supplement: Additional file 6 — Phylogenetic trees for T. reesei CAZymes. Trees are constructed from the protein clusters of 49 fungi including T. reesei CAZymes. Proteins are named with an uniprot protein identifier which is preceded by a code that specifies the species (Additional file 5). [file 1475-2859-11-134-S6.zip › Additional file 5/TRIRE0108346.eps.png]

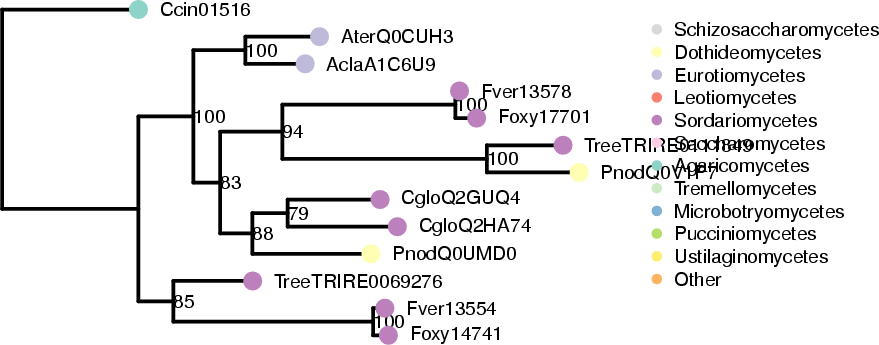

Supplement: Additional file 6 — Phylogenetic trees for T. reesei CAZymes. Trees are constructed from the protein clusters of 49 fungi including T. reesei CAZymes. Proteins are named with an uniprot protein identifier which is preceded by a code that specifies the species (Additional file 5). [file 1475-2859-11-134-S6.zip › Additional file 5/TRIRE0111849.eps.png]

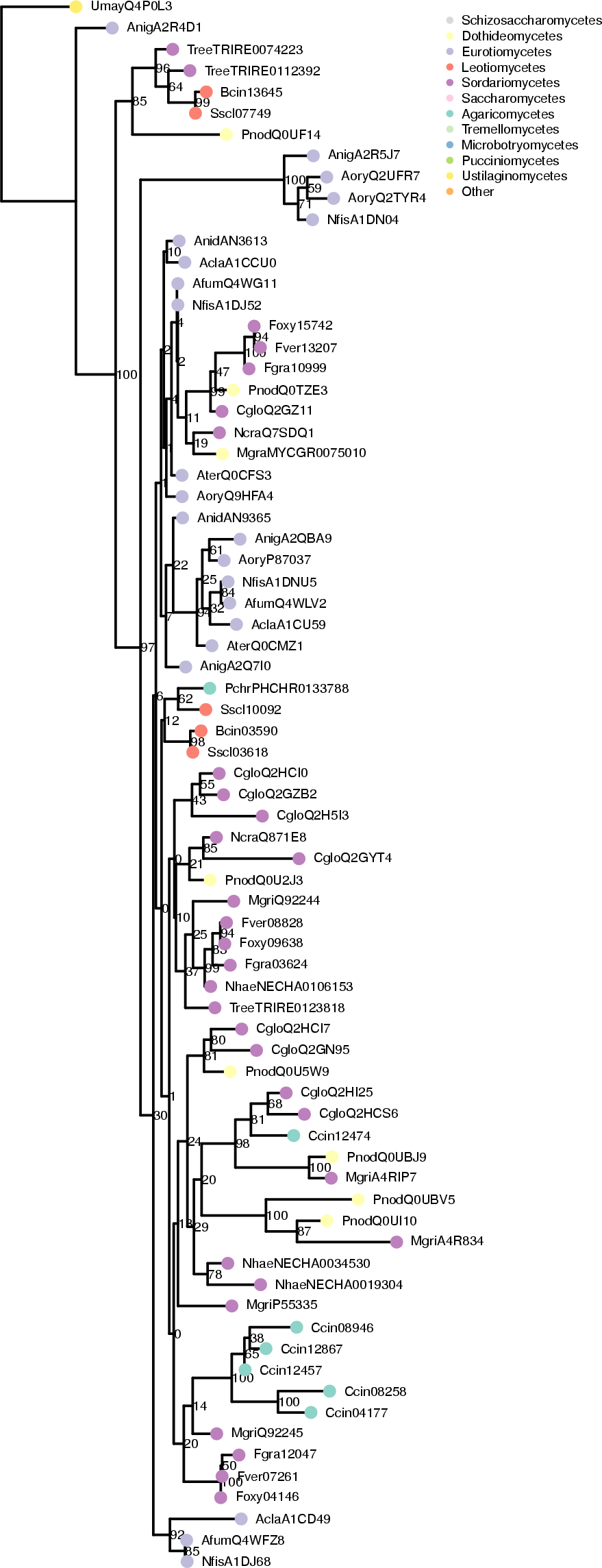

Supplement: Additional file 6 — Phylogenetic trees for T. reesei CAZymes. Trees are constructed from the protein clusters of 49 fungi including T. reesei CAZymes. Proteins are named with an uniprot protein identifier which is preceded by a code that specifies the species (Additional file 5). [file 1475-2859-11-134-S6.zip › Additional file 5/TRIRE0112392.eps.png]

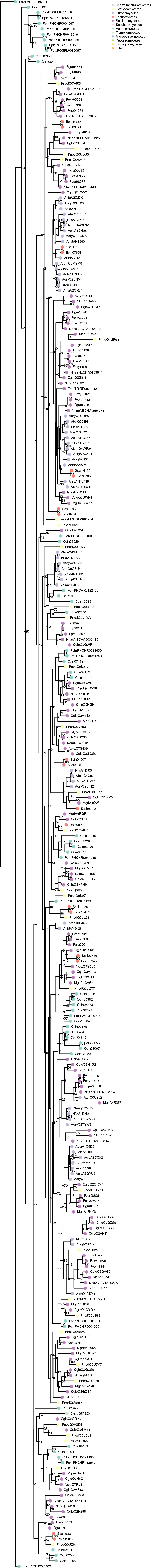

Supplement: Additional file 6 — Phylogenetic trees for T. reesei CAZymes. Trees are constructed from the protein clusters of 49 fungi including T. reesei CAZymes. Proteins are named with an uniprot protein identifier which is preceded by a code that specifies the species (Additional file 5). [file 1475-2859-11-134-S6.zip › Additional file 5/TRIRE0120961.eps.png]

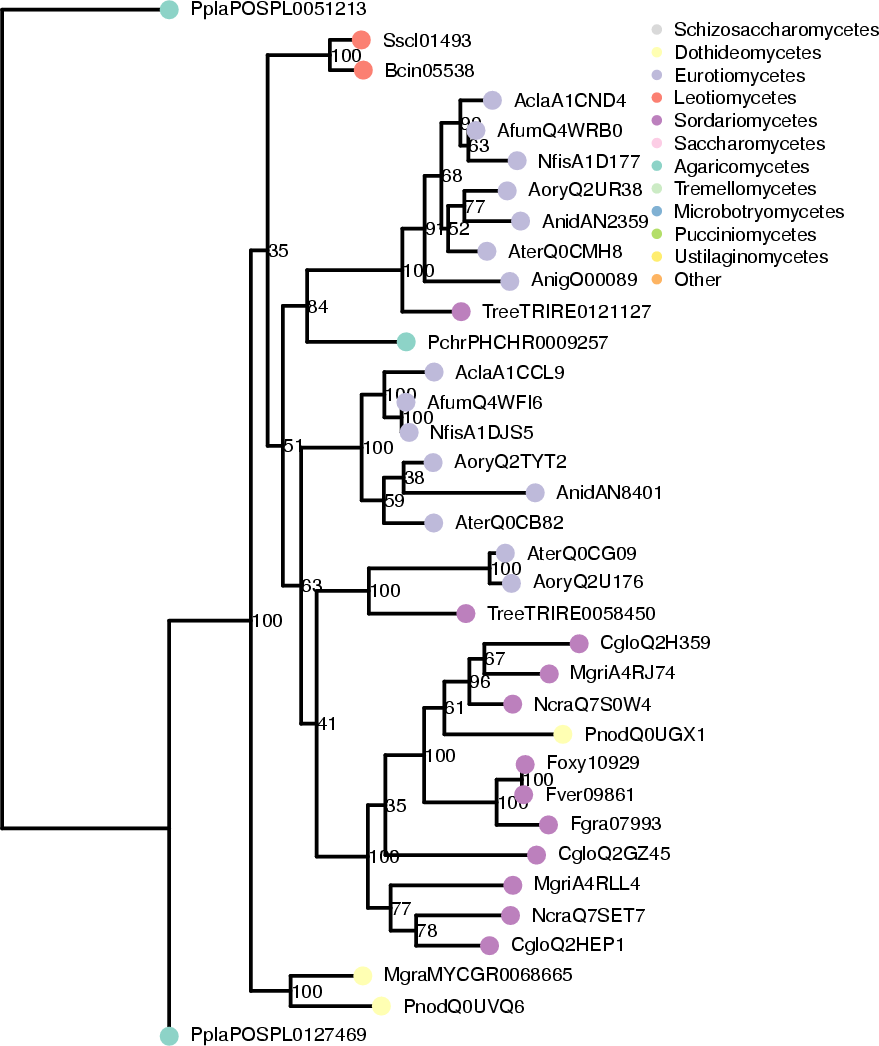

Supplement: Additional file 6 — Phylogenetic trees for T. reesei CAZymes. Trees are constructed from the protein clusters of 49 fungi including T. reesei CAZymes. Proteins are named with an uniprot protein identifier which is preceded by a code that specifies the species (Additional file 5). [file 1475-2859-11-134-S6.zip › Additional file 5/TRIRE0121127.eps.png]

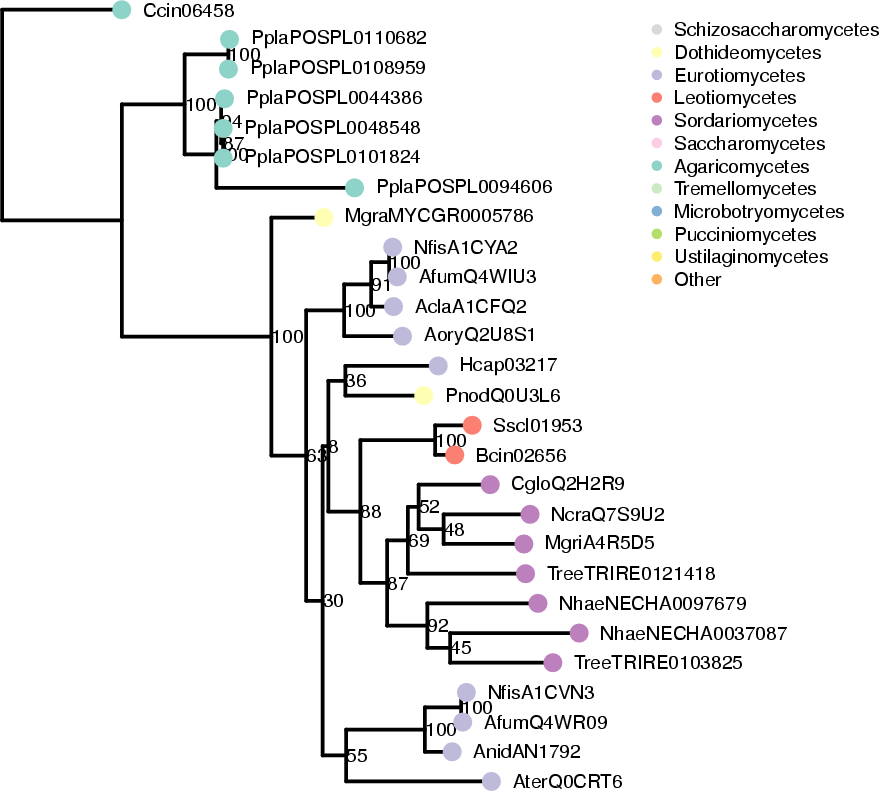

Supplement: Additional file 6 — Phylogenetic trees for T. reesei CAZymes. Trees are constructed from the protein clusters of 49 fungi including T. reesei CAZymes. Proteins are named with an uniprot protein identifier which is preceded by a code that specifies the species (Additional file 5). [file 1475-2859-11-134-S6.zip › Additional file 5/TRIRE0121418.eps.png]

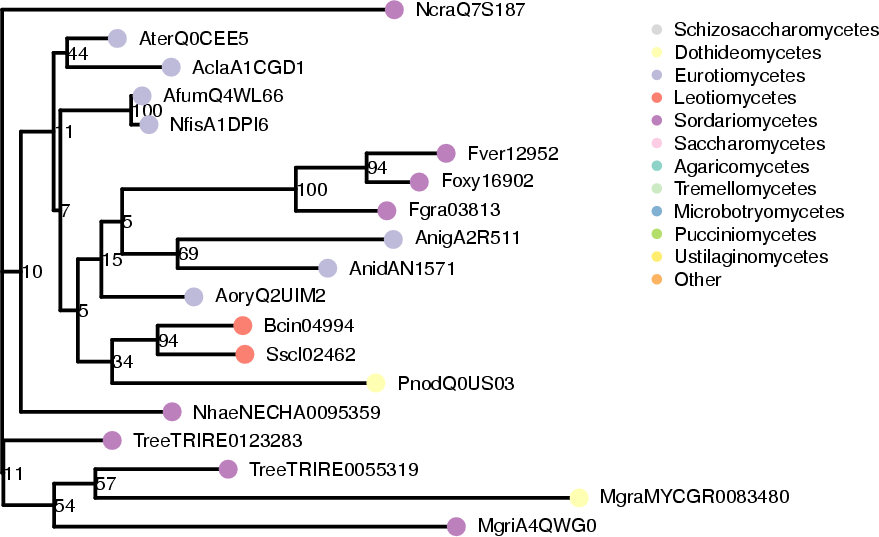

Supplement: Additional file 6 — Phylogenetic trees for T. reesei CAZymes. Trees are constructed from the protein clusters of 49 fungi including T. reesei CAZymes. Proteins are named with an uniprot protein identifier which is preceded by a code that specifies the species (Additional file 5). [file 1475-2859-11-134-S6.zip › Additional file 5/TRIRE0123283.eps.png]

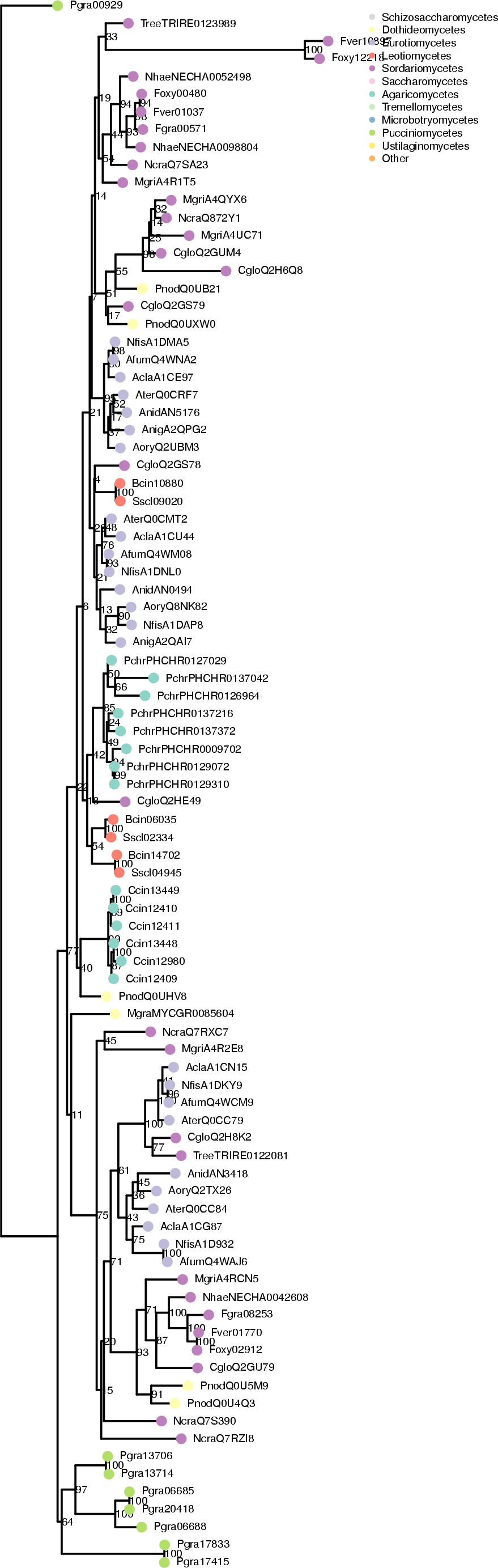

Supplement: Additional file 6 — Phylogenetic trees for T. reesei CAZymes. Trees are constructed from the protein clusters of 49 fungi including T. reesei CAZymes. Proteins are named with an uniprot protein identifier which is preceded by a code that specifies the species (Additional file 5). [file 1475-2859-11-134-S6.zip › Additional file 5/TRIRE0123989.eps.png]

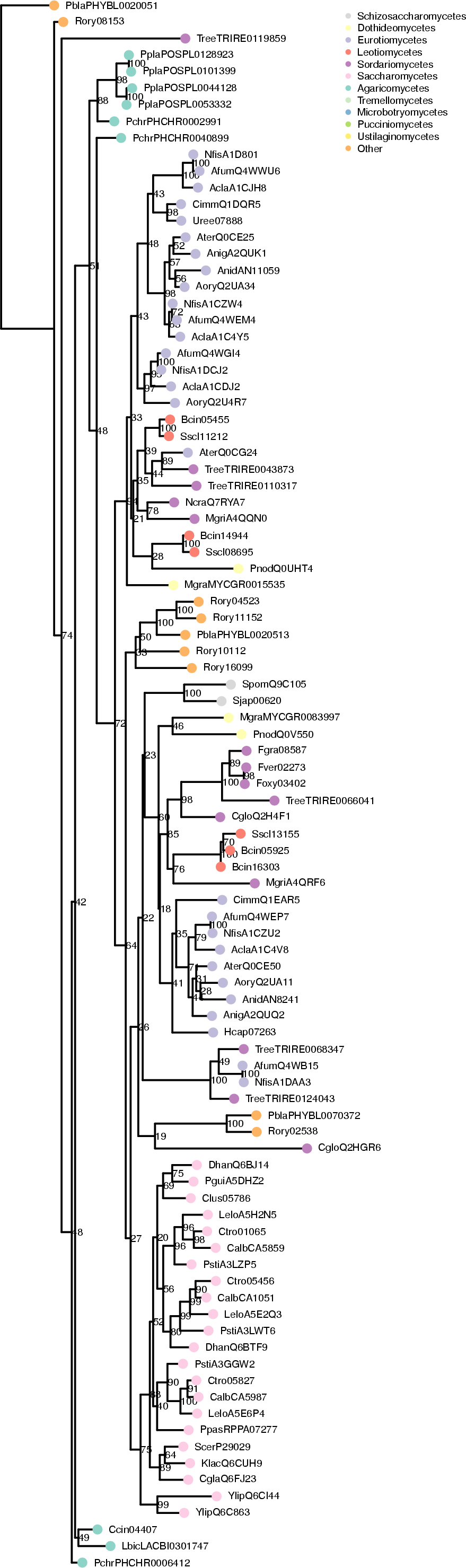

Supplement: Additional file 6 — Phylogenetic trees for T. reesei CAZymes. Trees are constructed from the protein clusters of 49 fungi including T. reesei CAZymes. Proteins are named with an uniprot protein identifier which is preceded by a code that specifies the species (Additional file 5). [file 1475-2859-11-134-S6.zip › Additional file 5/TRIRE0124043.eps.png]

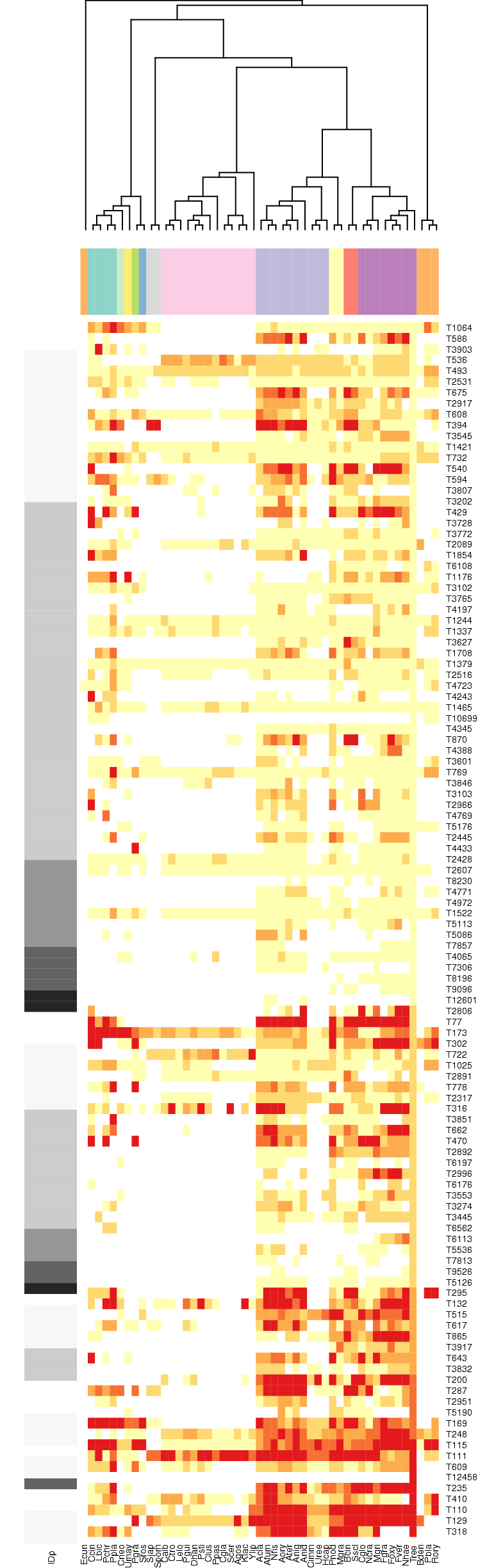

Supplement: Additional file 7 — Heatmap comparing the protein cluster content of different fungi. Each row is a protein cluster (marked with T and a number) and each column is a fungal species. The colouring of the cells is proportional to the count of proteins. A phylogram of the species is shown above the heatmap together with a colour bar coloured by the taxon of the species. Species abbreviations below the heatmap are explained in Additional file 5. On the left, the colour bar named IDp shows the identity percentage of the genes belonging to the same protein cluster. The darker the colour, the more identical the proteins are. [file 1475-2859-11-134-S7.png]

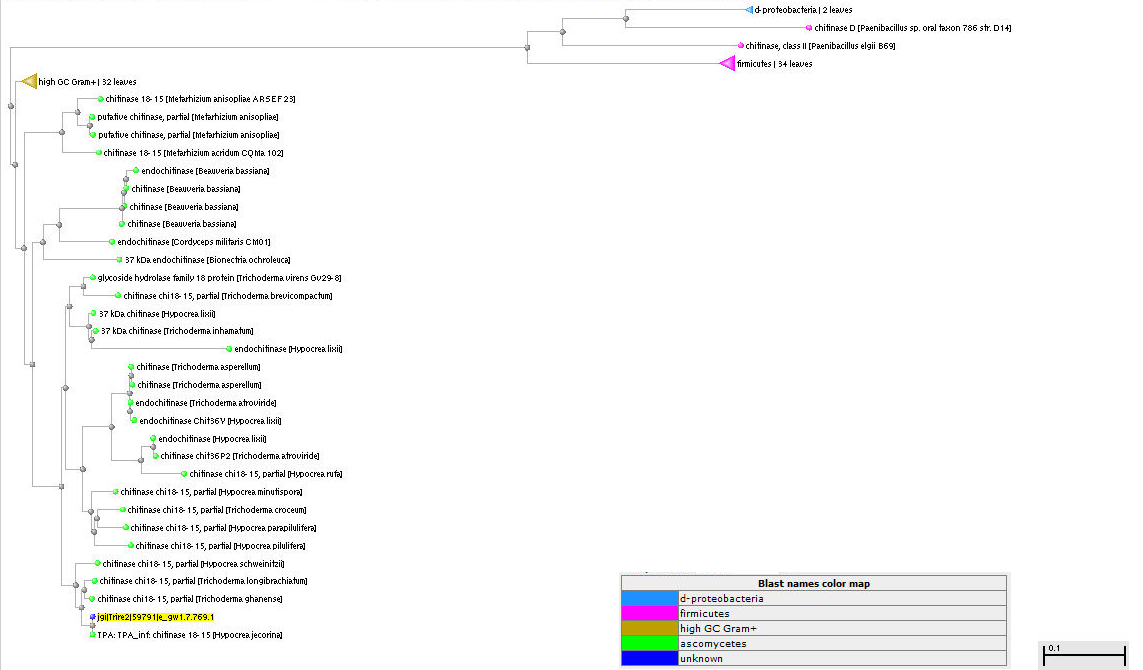

Supplement: Additional file 8 — Phylogeny of T. reesei CAZyme gene 59791. Tree was constructed from the results of blastp against the non-redundant proteinsequences database [82] using BLAST pairwise alignment. The tree method used was fast minimum evolution. Maximum sequence difference was 0.85 and distance model used was Grishin. [file 1475-2859-11-134-S8.png]

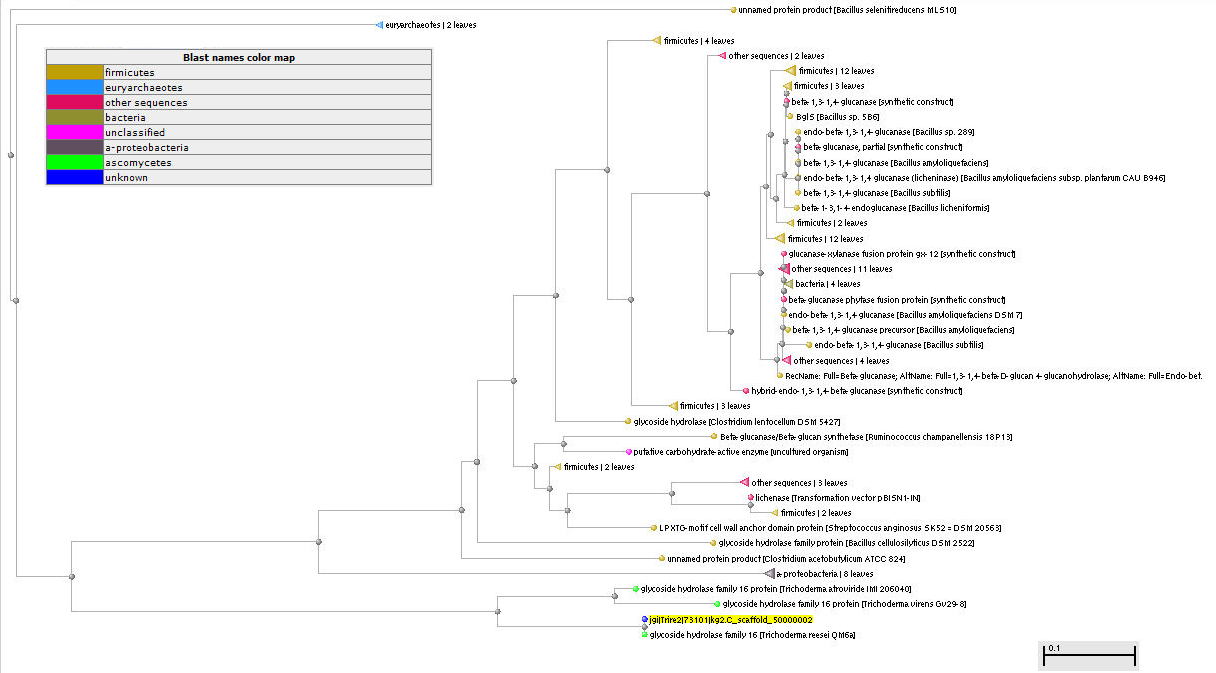

Supplement: Additional file 9 — Phylogeny of T. reesei CAZyme gene 73101. Tree was constructed from the results of blastp against the non-redundant proteinsequences database [82] using BLAST pairwise alignment. The tree method used was fast minimum evolution. Maximum sequence difference was 0.85 and distance model used was Grishin. [file 1475-2859-11-134-S9.png]

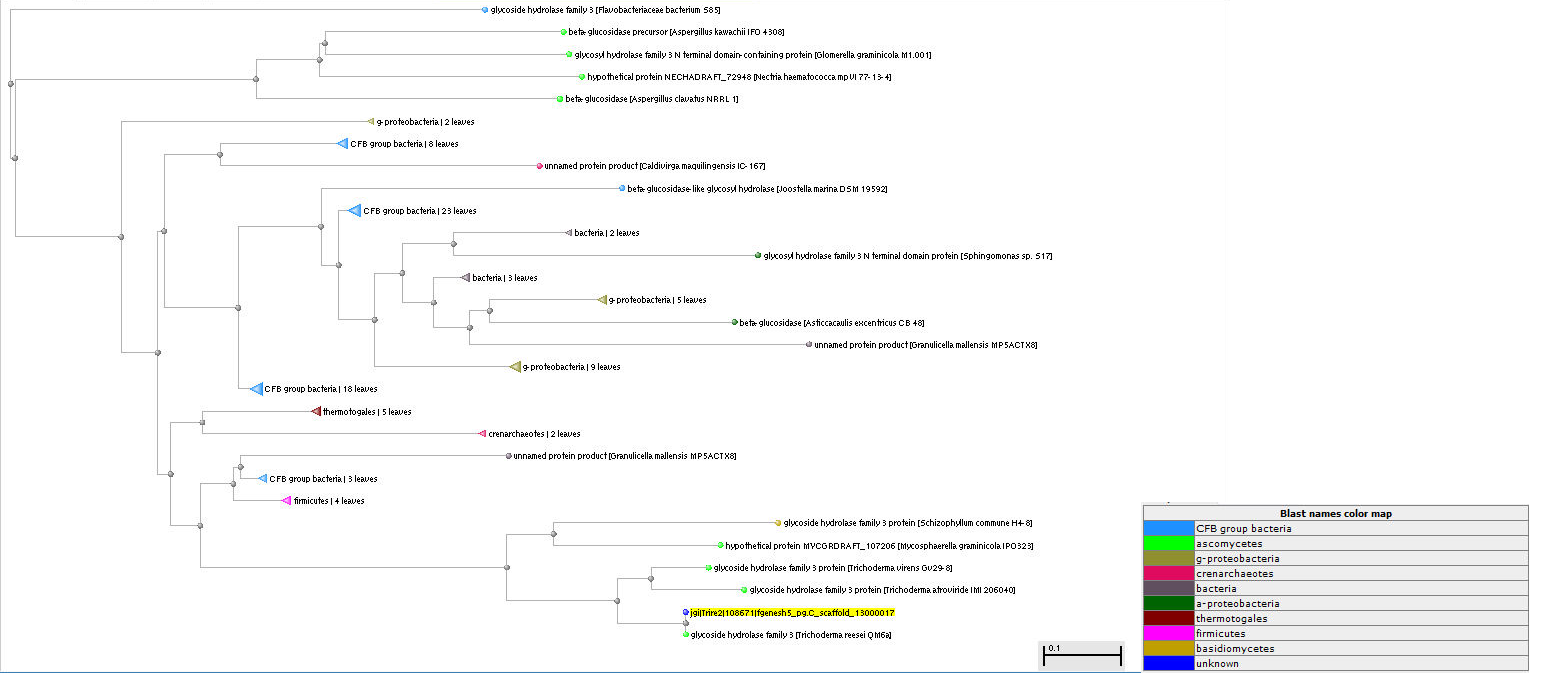

Supplement: Additional file 10 — Phylogeny of T. reesei CAZyme gene 108671. Tree was constructed from the results of blastp against the non-redundant proteinsequences database [82] using BLAST pairwise alignment. The tree method used was fast minimum evolution. Maximum sequence difference was 0.85 and distance model used was Grishin. [file 1475-2859-11-134-S10.png]
