# Supplementary material for: Re-annotation of the CAZy genes of Trichoderma reesei and transcription in the presence of lignocellulosic substrates
Source: Microb Cell Fact. 2012 Oct 4;11:134. doi: 10.1186/1475-2859-11-134 (PMC3526510; doi:10.1186/1475-2859-11-134)
Supplement: Additional file 12 — Comparison of relative transcript signals obtained using microarray or qPCR detection. Pre-processed and normalised microarray signals (log2 scale) were plotted against the relative expression signals obtained using qPCR analysis of the same samples (shown as -ΔCp, normalised using the signals of gpd1). Expression data of the genes xyn4, xyn3, egl2, cel61b, bxl1, egl1, abf1, xyn2, cbh1, swo1, cel3d, cbh2, axe1, xyn1 and cel3c were combined and plotted. [file 1475-2859-11-134-S12.pdf]

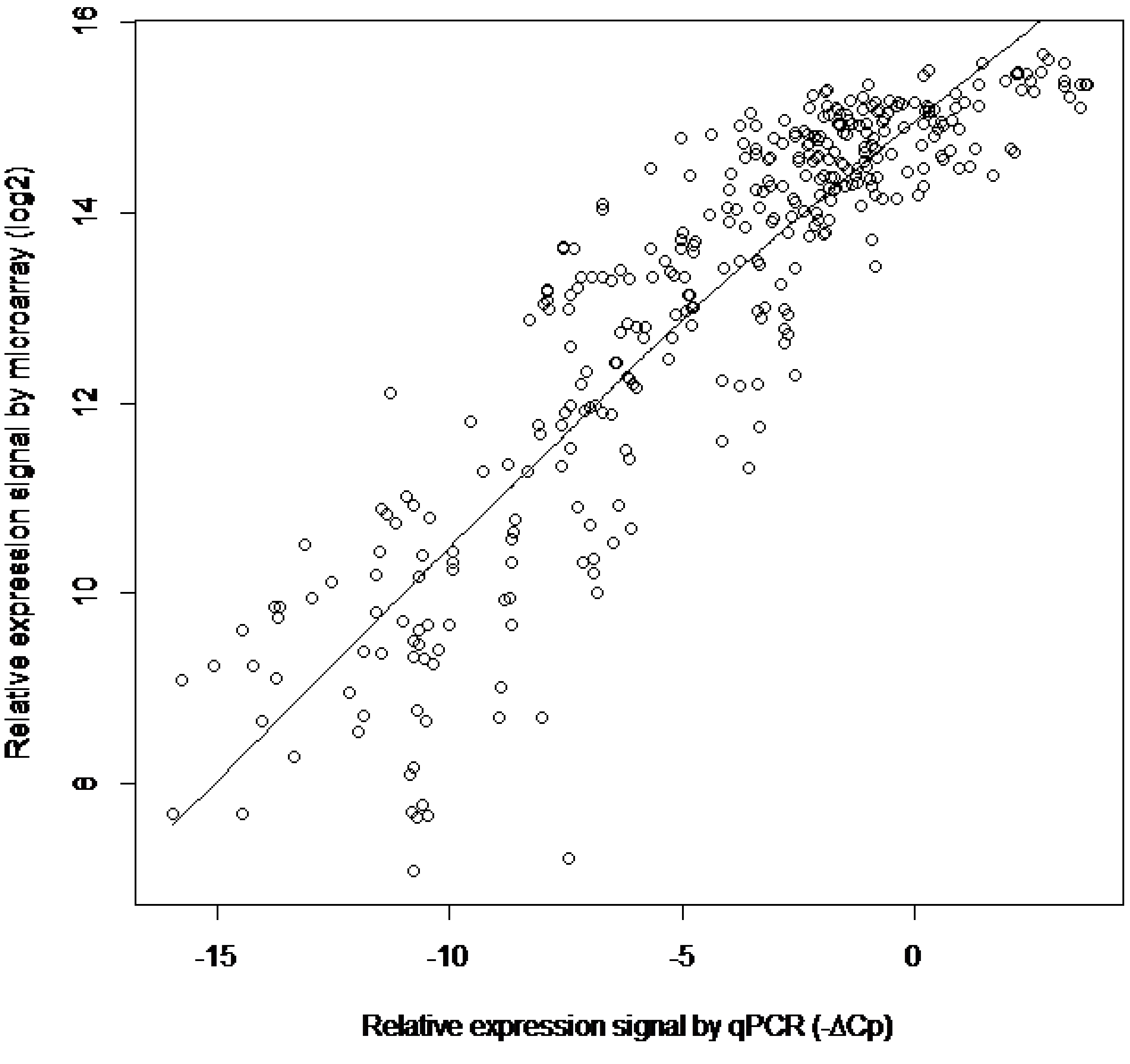

Additional file 12. Comparison of relative transcript signals obtained using microarray or qPCR detection. Pre-processed and normalised microarray signals (log2 scale) were plotted against the relative expression signals obtained using qPCR analysis of the same samples (shown as -ΔCp, normalised using the signals of gpd1). Expression data of the genes *xyn4*, *xyn3*, *egl2*, *cel61b*, *bxl1*, *egl1*, *abf1*, *xyn2*, *cbh1*, *swo1*, *cel3d*, *cbh2*, *axe1*, *xyn1* and *cel3c* were combined and plotted.
